# Improved CAR internalization and recycling through transmembrane domain optimization reduces CAR-T cytokine release and exhaustion

**DOI:** 10.3389/fimmu.2025.1531344

**Published:** 2025-03-27

**Authors:** Shufeng Xie, Jinlan Long, Ruiheng Wang, Rufang Xiang, Huajian Xian, Yixin Wang, Weiyu Dou, Wenjie Zhang, Dan Li, Ting Kang, Zhihong Chen, Chunjun Zhao, Zhenshu Xu, Han Liu

**Affiliations:** ^1^ Shanghai Institute of Hematology, State Key Laboratory of Medical Genomics, National Research Center for Translational Medicine at Shanghai, Ruijin Hospital, Shanghai Jiao Tong University School of Medicine, Shanghai, China; ^2^ School of Life Sciences and Biotechnology, Shanghai Jiao Tong University, Shanghai, China; ^3^ Fujian Institute of Hematology, Fujian Provincial Key Laboratory on Hematology, Fujian Medical University Union Hospital, Fuzhou, China; ^4^ Department of General Practice, Ruijin Hospital, Shanghai Jiao Tong University School of Medicine, Shanghai, China; ^5^ Emergency department, Chinese PLA Southern Theater Command General Hospital, Guangzhou, China; ^6^ Department of Oncology, Xin Hua Hospital, School of Medicine, Shanghai Jiao Tong University, Shanghai, China

**Keywords:** car-t, transmembrane domain, internalization, recycling, exhaustion, cytokine release

## Abstract

**Background:**

Anti-CD19 chimeric antigen receptor T (CAR-T) cell therapy has proven effective for treating relapsed or refractory acute B cell leukemia. However, challenges such as cytokine release syndrome, T cell dysfunction, and exhaustion persist. Enhancing CAR-T cell efficacy through changing CAR internalization and recycling is a promising approach. The transmembrane domain is the easiest motif to optimize for modulating CAR internalization and recycling without introducing additional domains, and its impact on CAR internalization and recycling has not yet been thoroughly explored. In this study, we aim to enhance CAR-T cell function by focusing on the solely transmembrane domain design.

**Methods:**

Utilizing plasmid construction and lentivirus generation, we get two different transmembrane CAR-T cells [19CAR-T(1a) and 19CAR-T(8α)]. Through co-culture with tumor cells, we evaluate CAR dynamic change, activation levels, exhaustion markers, mitochondrial function, and differentiation in both CAR-T cells. Furthermore, immunofluorescence microscopy analysis is performed to reveal the localization of internalized CAR molecules. RNA sequencing is used to detect the transcriptome of activated CAR-T cells. Finally, a mouse study is utilized to verify the anti-tumor efficacy of 19CAR-T(1a) cells *in vivo*.

**Results:**

Our findings demonstrate that 19CAR-T(1a) has lower surface CAR expression, faster internalization, and a higher recycling rate compared to 19CAR-T(8α). Internalized 19CAR(1a) co-localizes more with early and recycling endosomes, and less with lysosomes than 19CAR(8α). These features result in lower activation levels, less cytokine release, and reduced exhaustion markers in 19CAR-T(1a). Furthermore, CAR-T cells with CD1a transmembrane domain also exhibit a superior anti-tumor ability and reduced exhaustion *in vivo*.

**Conclusion:**

Overall, we demonstrate that the transmembrane domain plays a critical role in CAR-T cell function. An optimized transmembrane domain can alleviate cytokine release syndrome and reduce CAR-T cell exhaustion, providing a direction for CAR design to enhance CAR-T cell function.

## 1 Introduction

Anti-CD19 chimeric antigen receptor T (CAR-T) cell therapy has been demonstrated to effectively treat relapsed or refractory acute B cell leukemia (1). However, there are still several limitations to this therapy, such as the poor outcomes in solid tumors (2), cytokine release syndrome (CRS), immune effector cell-associated neurotoxicity syndrome (3, 4), and T cell dysfunction and exhaustion (5, 6). To address these limitations, strategies such as CAR structure optimization, cytokine co-expression, drug combination therapies, and dual antigen targeting have been explored (5, 7). Among these, CAR structure design appeals to much attention. Studies have shown that surface CAR levels influence CAR-T cytotoxicity (8–10), making it crucial to control surface CAR expression. Optimization strategies, such as modifying CAR internalization and recycling by incorporating the CTLA-4 tail (11) or enhancing CAR recycling by preventing CAR ubiquitination (12), have been demonstrated to improve CAR-T cell anti-tumor immunity. These strategies highlight the potential of enhancing CAR-T cell function by modulating CAR internalization and recycling. Given the CAR structure, which includes a single-chain variable fragment, hinge, transmembrane domain (TMD), costimulatory domain, and CD3ζ signaling domain, the TMD emerges as the easiest motif to optimize for modulating CAR internalization and recycling without introducing additional domains.

The hinge and TMD have been shown to influence CAR-T cell performance. For example, CAR-T cells with CD8α hinge and TMD produce lower cytokine levels and exhibit less activation-induced cell death compared to those with CD28 hinge and TMD (13). However, CD28 TMD modulates CAR-T cell activities by engaging endogenous CD28, revealing the difference between CD8α TMD and CD28 TMD (14). Furthermore, CAR with CD3ζ TMD forms a complex with endogenous CD3ζ enhancing CAR-Jurkat cells function (15). Additionally, CAR-T cells with ICOS hinge and TMD exhibit superior antitumor effects (16). The length change of hinge and TMD also shows different outcomes on CAR-T cells (17). These studies show the vital role of TMD in CAR-T cell functions. However, none have specifically examined the impact of the CAR TMD on internalization and recycling. Therefore, we aim to investigate whether modifying the TMD alone can influence CAR internalization and recycling to enhance CAR-T cell function. Here, we choose the CD1a TMD as our candidate. CD1a is a protein that surveys the endocytic pathway to deliver lipid antigens (18), and the study shows that CD1a TMD controls the endocytosis of CD1a protein (19). Thus, it provides a possibility to achieve CAR internalization and recycling by incorporating the CD1a TMD into the CAR molecule.

In this study, we constructed CAR-T cells with CD1a TMD and demonstrated that CD1a TMD CAR-T cells exhibited lower surface CAR levels, faster internalization and recycling rate, lower activation levels, less cytokine release and reduced exhaustion markers compared to CAR-T cells with CD8α TMD. Furthermore, CD1a TMD CAR-T cells have a superior anti-tumor ability *in vivo*. This study demonstrated that the solely TMD design of the CAR molecule could effectively influence CAR internalization and recycling, and was an addition to the cognition that TMD of CAR impacted CAR-T cells efficacy. In addition, this study also provides a direction that optimizing the TMD alone could be an effective strategy to alleviate CRS and reduce exhaustion for enhancing CAR-T cell functions.

## 2 Materials and methods

### 2.1 Cell lines and cell culture

Human cell lines SEM, Jurkat, and K562 were purchased from DSMZ. Cells were cultured in RPMI 1640 containing 10% FBS, at 37°C with 5% CO_2_. Peripheral blood mononuclear cells were obtained from healthy donors. CD3^+^ T cells were isolated using EasySep™ Human T Cell Enrichment Kit (STEMCELL Technologies, 19051) and then cultured in CTS™ AIM V™ SFM medium (Thermo Fisher Scientific, 0870112DK) containing 5% human serum AB (GEMINI, 100-512), 1% GlutaMAX (Thermo Fisher Scientific, 35050061), and 100 U/mL human IL-2 (PeproTech, 200-02). The CD3^+^ T cells were activated by Dynabeads™ Human T-Activator CD3/CD28 (Thermo Fisher Scientific, 11131D).

### 2.2 CAR expression and CD19 detection

The surface CAR was detected by using APC or Brilliant Violet 421™ anti-FLAG Tag Antibody (Biolegend, 637308/637322). The total CAR was staining intracellular FLAG expression by APC or Brilliant Violet 421™ anti-FLAG Tag Antibody after using BD Cytofix/Cytoperm Buffer System (BD, 554714). The CD19 protein was detected by APC anti-human CD19 Antibody (BD, 555415). Data produced by the flow cytometer (BD LSRFortessa™ X-20) were analyzed using the FlowJo software.

### 2.3 Lentivirus-based generation of CAR-T and cell lines

Lentivirus plasmids pHR-FLAG-Anti-CD19 single-chain variable fragment (FMC63)-CD8 hinge-CD8α or CD1a transmembrane domain-CD28 and 4-1BB costimulatory domain-CD3ζ-copGFP (pHR-FMC63-CD8α/1a-CAR) was generated by inserting CAR sequence [generated according to the patent previously reported (20)] into the pHR plasmids (Addgene). The pCDH-CD19-mRuby2 plasmid was generated by inserting CD19 and mRuby2 sequences into the pCDH lentiviral vector (System Biosciences). The pCDH-Luciferase-mRuby2 plasmid was generated by inserting Luciferase and mRuby2 sequences into the pCDH lentiviral vector. For CAR-T cells, human CD3^+^ T cells activated for 72 h were collected and resuspended with concentrated lentiviruses (pHR-FMC63-CD8α/1a-CAR, MOI=15). 0.5 million cells were plated on a 24-well plate and spun at 1200 g for 1 h at 37°C. The virus supernatant was aspirated and replaced with fresh medium after 24 h infection. The CAR-T cells were checked 3 days after infection by flow cytometry. The endotoxin levels in CAR-T cells were within permissible ranges (Yeasen, 36723ES). And there was no mycoplasma contamination (Yeasen, 40601ES). For CAR-Jurkat cells, after infection, cells were sorted according to surface CAR levels to get different surface CAR expression cells. For SEM-mRuby2-Luciferase and CD19-K562 cells, after infection (pCDH-Luciferase-mRuby2 and pCDH-CD19-mRuby2, respectively), positive cells were sorted according to mRuby2.

### 2.4 CAR-T/CAR-Jurkat cytotoxicity assay

The target cells (SEM or CD19-K562) were stained with CellTrace™ Violet/Yellow/Far Red (Thermo Fisher Scientific, C34571/C34573/C34572) as wanted. Then CAR-T/CAR-Jurkat cells were co-cultured with target cells at different E/T ratios for the indicated time, and the apoptosis of target cells was detected using the Annexin V-APC or Annexin V-Pacific Blue Apoptosis Detection Kit (BioLegend, 640930/640926). Data produced by the flow cytometer were analyzed using the FlowJo software.

### 2.5 CAR internalization assay

For antibody-based assay (11), CAR-T or CAR-Jurkat cells were stained with Brilliant Violet 421™ anti-FLAG Tag Antibody at 4°C for 30 min and washed with PBS. Then these cells were incubated at 37°C for 0, 1, 2, or 4 h in the medium. Next, these cells were stained with Alexa Fluor^®^ 647 Goat anti-rat IgG (minimal x-reactivity) Antibody (BioLegend, 405416) at 4°C for 30 min. The decrease in the percentage of cells staining positive for the secondary antibody was quantified by flow cytometry as an indication of the internalization rate. For Brefeldin A-based assay (12), CAR-T cells were treated with 10 μM Brefeldin A (Topscience, T6062) at 37°C for 0, 1, 2, or 4 h, and then the surface CAR was detected by the anti-FLAG antibody. CAR-Jurkat cells were co-cultured with SEM at a 1:1 ratio for 0, 1, 2, or 4 h with the presence of 10 μM Brefeldin A, and the surface CAR was detected by anti-FLAG antibody.

### 2.6 CAR recycling assay

For antibody-based assay (11), CAR-T or CAR-Jurkat cells were stained with Brilliant Violet 421™ anti-FLAG Tag Antibody at 37°C for 30 min and washed with PBS. Then the cells were stained with Alexa Fluor^®^ 647 Goat anti-rat IgG (minimal x-reactivity) Antibody at 4°C for 30 min (baseline) or at 37°C for 30 min or 60 min. The increase in the percentage of cells staining positive for the secondary antibody was quantified by flow cytometry as an indication of recycling.

### 2.7 CAR degradation assay

CAR-T cells alone or co-cultured with SEM at a 1:1 ratio was treated with 50 µg/mL cycloheximide (MedChemExpress, HY-12320) at 37°C for 0, 1, 2, or 4 h. The total CAR was staining intracellular FLAG expression by anti-FLAG Tag Antibody after using BD Cytofix/Cytoperm Buffer System. The decrease in the percentage of total CAR was quantified by flow cytometry as an indication of degradation rate.

### 2.8 Immunofluorescence

CAR-T or CAR-Jurkat cells were co-cultured with SEM cells at a 1:1 ratio for 4 h, and the cells were then stained with mouse anti-FLAG antibody (Sigma, F1804) in combination with one of the following rabbit antibodies: anti-human Rab5 (CST, 3547P), anti-human Rab11 (Proteintech, 15903-1-AP), or anti-human LAMP1 (Abgent, AP1823a-ev). Then Goat anti-Mouse IgG (H+L) Secondary Antibody, DyLight™ 650 (Thermo Fisher Scientific, SA5-10174) was used to stain mouse anti-FLAG antibody, and Goat anti-Rabbit IgG (H+L) Highly Cross-Adsorbed Secondary Antibody, Alexa Fluor™ 568 (Thermo Fisher Scientific, A11036) was used to stain rabbit anti-human Rab5, Rab11, or LAMP1. Positive CAR-T cells were identified by staining with the anti-FLAG antibody.

### 2.9 RNA-seq analysis

CAR-T cells were sorted after co-culture with SEM cells at 1:1 for 24 h. The mRNA-Seq library was constructed and then sequenced using the MGISEQ-2000RS (BGI Technology Service). Differentially expressed genes were selected based on |log2FoldChange| > 1 and Q-value < 0.05.

### 2.10 CAR-T activation and exhaustion detection

CAR-T cells were co-cultured with target cells for the indicated time. Then CD69 was detected by APC anti-human CD69 antibody (BioLegend, 310910) and CD25 was detected by PE anti-human CD25 antibody (BioLegend, 302606) as an indication of CAR-T activation. For exhaustion detection, CAR-T cells were co-cultured with target cells (SEM or CD19-K562) at the indicated E/T ratio for 3 days, and exhaustion markers on CAR-T cells were detected by APC anti-human CD279 (PD-1) Antibody (BioLegend, 367405), PE anti-human CD223 (LAG-3) Antibody (BioLegend, 369305), and PerCP/Cyanine5.5 anti-human CD366 (TIM-3) Antibody (BioLegend, 345015).

### 2.11 CAR-T cytokines release detection

CAR-T cells were co-cultured with SEM cells at a 1:1 ratio for 24 h, and the supernatant was collected for detection of cytokines by LEGENDplex™ Human CD8/NK Panel (BioLegend, 741065).

### 2.12 Mitochondrial function assay

CAR-T cells alone or co-cultured with SEM at a 1:1 ratio for the indicated time, and 200 nM TMRE (MedChemExpress, HY-D0985A) was used to detect CAR-T cells mitochondrial activity, 200 nM MitoTracker Deep Red FM (Beyotime Biotechnology, C1032) was used to detect CAR-T cells mitochondrial mass, and 5 μM MitoSOX Red (MedChemExpress, HY-D1055) was used to detect CAR-T cells mitochondrial ROS.

### 2.13 CAR-T differentiation measurement

CAR-T cells were co-cultured with SEM cells at 1:1 for 3 days, and CD62L or CD45RA on CAR-T cells were detected by APC anti-human CD62L (BioLegend, 304809) or PE anti-human CD45RA (BioLegend, 304108) antibodies.

### 2.14 Mouse studies

NOD/SCID mice were purchased from Vital River Laboratory. For survival assay, 1 × 10^6^ SEM-mRuby2-Luciferase tumor cells were injected into mice (5 weeks, female) intravenously. Mice were allocated randomly into different experimental groups (3 groups [T, 19CAR-T(8α), 19CAR-T(1a)], n=4) and then intravenously administered 1 × 10^6^ CAR-T cells on the third day after injection of tumor cells. Tumor growth was monitored by the quantitative imaging system. For CAR-T cell detection *in vivo*, 1 × 10^6^ SEM-mRuby2-Luciferase tumor cells were injected into mice (5 weeks, female) intravenously. Mice were allocated randomly into different experimental groups (3 groups [T, 19CAR-T(8α), 19CAR-T(1a)], n=3) and then intravenously administered 1.5 × 10^6^ CAR-T cells on the 17th day after injection of tumor cells. Then mice were sacrificed on the third day after injection of CAR-T cells. The total cells in bone marrow were collected. First, the dead cells were stained by Fixable Viability Dye eFluo™ 780 (Thermo Fisher Scientific, 65-0865-14). Then the cells were treated by TruStain Fc™ PLUS (anti-mouse CD16/32) Antibody (BioLegend, 156604) on ice for 10 min. Finally, PerCP/Cyanine5.5 anti-human CD45 (BioLegend, 982312), Brilliant Violet 605™ anti-human TIM-3 (BioLegend, 345017), Brilliant Violet 785™ anti-human LAG-3 (BioLegend, 369321), and APC anti-human PD-1 antibodies were stained at 4°C for 30 min. Data produced by the flow cytometer (BD FACSymphony™ A3) were analyzed using the FlowJo software.

### 2.15 Statistical analysis

The two-tailed Student *t*-test was used to analyze the differences between the control and experimental groups. The log-rank Kaplan-Meier survival test was used to compare the survival distributions of the different treatment groups in an SEM-mRuby2-Luciferase xenograft mouse model. The Pearson correlation coefficient was calculated by ImageJ. The statistical significance level was calculated by GraphPad Prism indicated as * for P < 0.05, ** for P < 0.01, *** for P < 0.001, or **** for P < 0.0001, and the ns indicate no significant difference. Error bars reflect ± SD of three independent experiments.

## 3 Results

### 3.1 CD1a transmembrane CAR-T cells exhibit lower surface CAR levels

**Figure 1:**
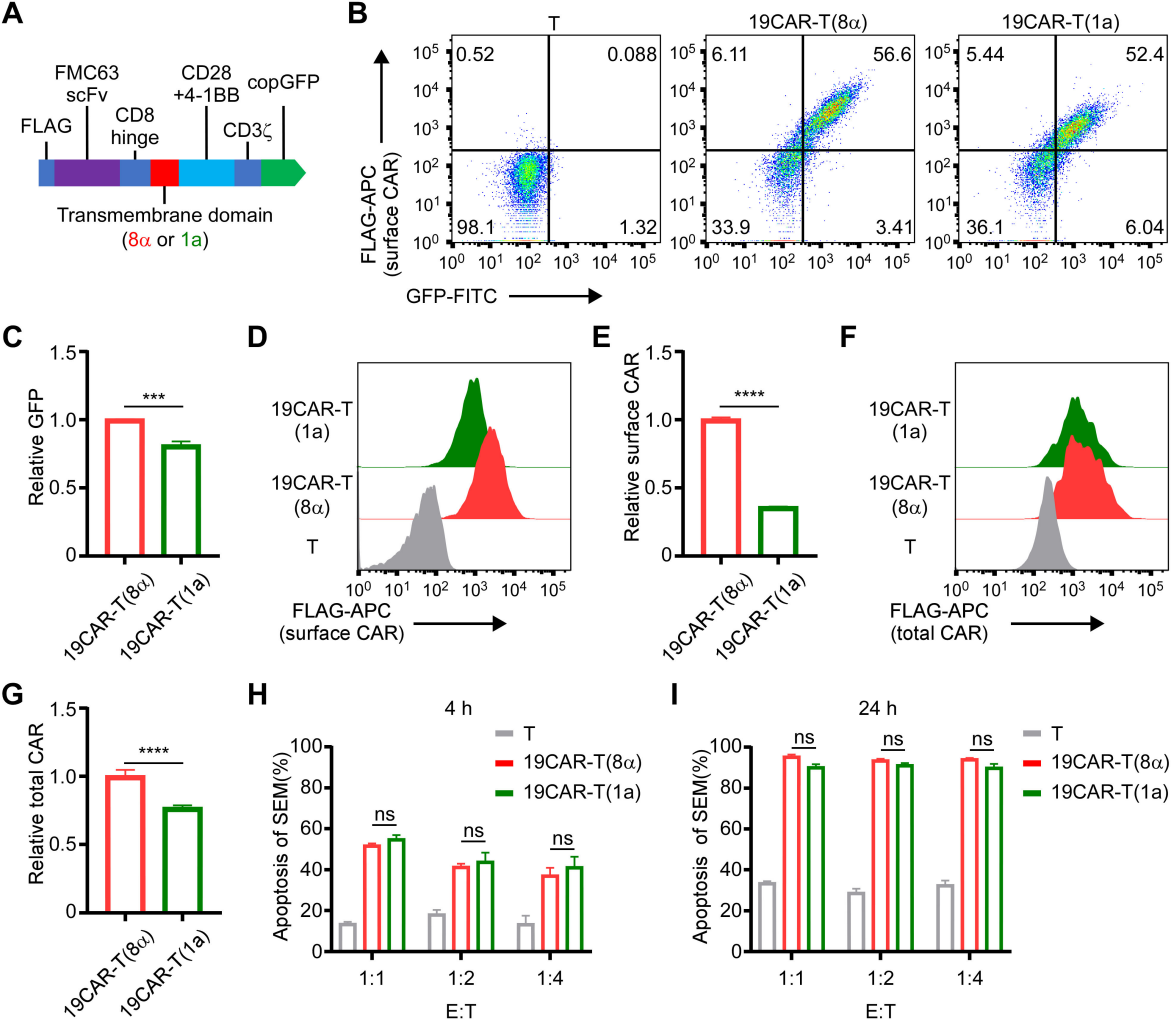
CD1a transmembrane CAR-T cells exhibit lower surface CAR levels. **(A)** The schematic of CAR structure. **(B)** The positive rate of CAR-T cells is indicated by GFP and anti-FLAG antibody. **(C)** The relative total CAR on CAR-T cells is indicated by GFP expression. Normalized to 19CAR-T(8α) GFP MFI. **(D)** The surface CAR expression on CAR-T cells is indicated by anti-FLAG antibody. **(E)** The relative surface CAR expression on CAR-T cells is indicated by anti-FLAG antibody. Normalized to 19CAR-T(8α) surface CAR. **(F)** The total CAR expression on CAR-T cells was indicated by staining intracellular FLAG expression with anti-FLAG antibody. **(G)** The relative total CAR expression on CAR-T cells is indicated by staining intracellular FLAG expression with anti-FLAG antibody. Normalized to 19CAR-T(8α) total CAR. **(H, I)** CAR-T cells were co-cultured with SEM cells at 1:1, 1:2 or 1:4 for 4 h **(H)** or 24 h **(I)**. The apoptosis of target cells was detected by using the Annexin V kit. Two-tailed Student *t*-test, *** for P < 0.001, **** for P < 0.0001, the ns indicate no significant difference. Error bars reflect ± SD of three independent experiments.

Here, we constructed a novel CAR incorporating the TMD from the CD1a molecule, differing from traditional CD8α transmembrane CAR (13, 21, 22). The other domains of the CAR were FLAG, CD19 single-chain variable fragment (FMC63), CD8 hinge, CD28 and 4-1BB costimulatory domain, CD3ζ signal domain, and copGFP (**Figure 1A**). We named these two different CAR-T 19CAR-T(8α) and 19CAR-T(1a), respectively. Both CAR-T cells were generated by infecting CD3^+^ T cells with the same multiplicity of infection (MOI = 15) (**Figure 1B**). Here, the CAR levels in 19CAR-T(1a) were nearly the same as those in 19CAR-T(8α) based on GFP signals, although 19CAR-T(1a) showed slightly lower levels (**Figure 1C**). However, we measured surface CAR on CAR-T cells using anti-FLAG antibody by flow cytometry and discovered that the CAR molecules on 19CAR-T(8α) cell surface were almost 3-fold than 19CAR-T(1a), showing a difference from GFP signal (**Figures 1D, E**). We hypothesized that this difference was due to the accumulation of CAR molecules in the cytoplasm of 19CAR-T(1a) cells rather than on the cell surface. To test this, intracellular flow cytometry was used to detect total CAR in CAR-T cells. The result showed that the total CAR in 19CAR-T(1a) was relatively high and consistent with the GFP signal (**Figures 1F, G**). These data indicated that 19CAR-T(1a) had low surface CAR levels, with a significant amount of CAR retained intracellularly. Since surface CAR expression has been shown to influence CAR-T cell cytotoxicity (8–10), we performed both short- and long-time co-culture of CAR-T cells with SEM tumor cells. Surprisingly, there was no significant difference in SEM cell apoptosis between these two CAR-T cells (**Figures 1H, I**). This suggests that despite the lower surface CAR expression, 19CAR-T(1a) cells possess sufficient cytotoxicity to kill tumor cells, comparable to 19CAR-T(8α) cells.

### 3.2 CD1a transmembrane CAR shows rapid internalization and recycling rate

**Figure 2:**
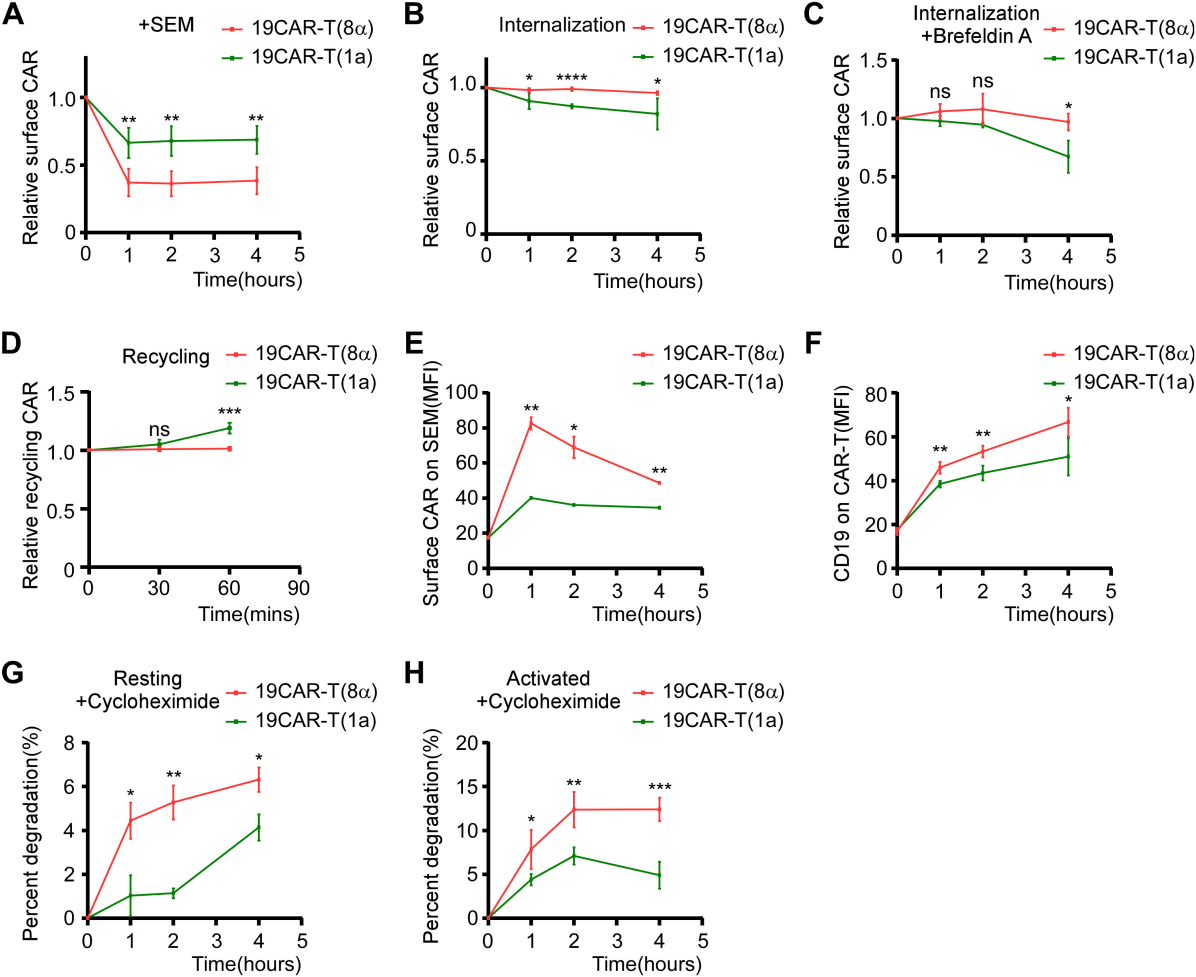
CD1a transmembrane CAR shows rapid internalization and recycling rate. **(A)** CAR-T cells were co-cultured with SEM cells at 1:1 for 1, 2, or 4 hThe surface CAR on CAR-T cells was detected by anti-FLAG antibody. Normalized to each CAR-T cell non-cocultured surface CAR, respectively. **(B)** Antibody-based assay for CAR internalization. **(C)** Brefeldin A-based assay for CAR internalization. CAR-T cells were treated with 10 μM Brefeldin A at 37°C for 0, 1, 2, or 4 h, then the surface CAR was detected by anti-FLAG antibody. Normalized to each CAR-T cell Brefeldin A non-treated surface CAR, respectively. **(D)** Antibody-based assay for CAR recycling. **(E)** CAR-T cells were co-cultured with SEM cells at 1:1 for 0, 1, 2, or 4 h The transferred surface CAR on SEM cells was detected by anti-FLAG antibody. **(F)** CAR-T cells were co-cultured with SEM cells at 1:1 for 0, 1, 2, or 4 h The transferred CD19 on CAR-T cells was detected by anti-CD19 antibody. **(G, H)** CAR-T cells **(G)** or CAR-T cells co-cultured with SEM at a 1:1 ratio **(H)** were treated with 50 µg/mL cycloheximide at 37°C for 0, 1, 2, or 4 h The total CAR was staining intracellular FLAG expression by anti-FLAG antibody after using BD Cytofix/Cytoperm Buffer System. The decrease in the percentage of total CAR on CAR-T cells was quantified by flow cytometry as an indication of degradation rate. Two-tailed Student *t*-test, * for P < 0.05, ** for P < 0.01, *** for P < 0.001, **** for P < 0.0001, the ns indicate no significant difference. Error bars reflect ± SD of three independent experiments.

According to the study that the CD1a TMD controls the endocytosis of CD1a protein (19), the exploration of dynamic change of CAR molecules on 19CAR-T(1a) is needed. First, CAR-T cells were co-cultured with SEM cells, and the surface CAR was detected. We observed that 19CAR(1a) decreased less than 19CAR(8α) (**Figure 2A**). Given the different expressions of surface CAR on these two CAR-T cells, the results may be influenced by baseline surface CAR levels. Therefore, we constructed 19CAR-Jurkat(8α) cell lines expressing high, middle, and low levels of CAR (named 19CAR-J(8α)^H^, 19CAR-J(8α)^M^, and 19CAR-J(8α)^L^), with 19CAR-J(8α)^M^ expressing surface CAR levels similar to 19CAR-J(1a) (**Supplementary Figures S1A, B**). Then the cytotoxicity of 19CAR-J(8α)^M^ and 19CAR-J(1a) was confirmed by detecting the apoptosis of SEM cells under different E/T ratios. The results reflected similar cytotoxicity between these two CAR-J cells, though 19CAR-J(1a) could induce slightly more tumor cell apoptosis under low E/T after 72 h of co-culture (**Supplementary Figure S1C**). Next, we employed these CAR-J cells to co-culture with SEM cells at 1:2 for 24 h and measured the surface CAR changes. We found that CAR levels decreased across all CAR-J cells, and 19CAR-J(8α)^H^ and 19CAR-J(8α)^M^ decreased more CAR molecule than 19CAR-J(8α)^L^, reflecting the surface CAR levels influence the statistical analysis of the decrease of CAR (**Supplementary Figures S2A, B**). Importantly, 19CAR-J(1a) exhibited a more significant decrease in surface CAR than 19CAR-J(8α)^M^ (**Supplementary Figures S2A, B**), and the result was confirmed by co-culture of SEM and 19CAR-J(8α)^M^ and 19CAR-J(1a) at different E/T ratios (**Supplementary Figures S2C–E**). There was a difference in the results between the CAR-J and CAR-T cells due to the different surface CAR expressions. Thus, to directly assess CAR internalization, we employed a previously reported assay (11). The results showed that 19CAR(1a) had a faster internalization rate than 19CAR(8α), both in CAR-T and CAR-J cells (**Figure 2B**; **Supplementary Figure S2F**). To further validate the conclusion, brefeldin A (BFA), a compound inducing fusion of early endosomes with the *trans*-Golgi network, was used to study the internalization of CAR (12). The results suggested that 19CAR(1a) had rapid internalization rate than 19CAR(8α) under BFA treatment (**Figure 2C**). What’s more, the same result could be seen in co-cultured CAR-J and SEM cells under BFA treatment (**Supplementary Figure S2G**). Besides the internalization of CAR, we also investigated the recycling rate of CAR molecules according to the method reported previously (11). The results indicated that 19CAR-T(1a) had a higher rate of CAR recycling compared to 19CAR-T(8α), and the same results could be seen on the CAR-J cells (**Figure 2D**; **Supplementary Figure S2H**). Collectively, these data suggest that the CD1a transmembrane CAR shows a rapid internalization and recycling rate.

### 3.3 Only a small amount of CAR or CD19 undergo trogocytosis

Interactions between cells, such as trogocytosis, have been shown to affect CAR-T cell function (23). Hence, we measured CAR and CD19 proteins on effector or target cells after co-culture. We found that SEM acquired a small amount of CAR molecules after co-culture with CAR-T cells, with 19CAR-T(8α) transferring more CAR proteins to SEM than 19CAR-T(1a) (**Figure 2E**; **Supplementary Figure S3A**). To confirm these results, we repeated the experiment using CAR-J cells. The results showed that 19CAR-J(8α)^H^ transferred more CAR proteins to SEM than 19CAR-J(8α)^M^, and there was no significant difference between 19CAR-J(8α)^M^ and 19CAR-J(1a), suggesting that surface CAR levels, rather than the TMD, influenced the CAR transfer (**Supplementary Figure S3B**). Although only a small amount of CAR was transferred, we explored whether this part of CAR will affect CAR-J cell cytotoxicity. First, we sorted SEM cells after co-culture with 19CAR-J(8α)^H^ cells at 1:1 for 24 h and tracked the persistence of transferred CAR on the SEM surface over time. We found that the transferred CAR could not sustain on the SEM surface and almost disappeared after 72 h (**Supplementary Figure S3C**). Furthermore, when the sorted SEM cells were co-cultured again with 19CAR-J(8α)^H^ cells, there was no significant difference in apoptosis between SEM, SEM^J^, and SEM^19CAR-J(8α)H^ cells, suggesting that the small amount of transferred CAR on SEM cells had no impact on its apoptosis induced by CAR-J cells (**Supplementary Figures S3D, E**). Similarly, we tracked the levels of CD19 proteins on CAR-T cells over time and found that 19CAR-T(8α) cells acquired slightly more CD19 proteins than 19CAR-T(1a) (**Figure 2F**). However, only a small amount of CD19 was transferred compared to CD19 on SEM cells (**Supplementary Figure S3F**). Co-culture experiments with 19CAR-J(8α)^M^ and 19CAR-J(1a) cells showed that CD19 levels on SEM cells decreased significantly, consistent with the report that anti-CD19 CAR induced CD19 internalization (24), and there was no obvious difference between 19CAR-J(8α)^M^ and 19CAR-J(1a) (**Supplementary Figure S3G**). These data reveal that only a small amount of CD19 or CAR is transferred to effector or target cells, and the majority of CAR molecules undergo internalization.

### 3.4 Internalized CD1a transmembrane CAR co-localizes more with early and recycling endosomes

**Figure 3:**
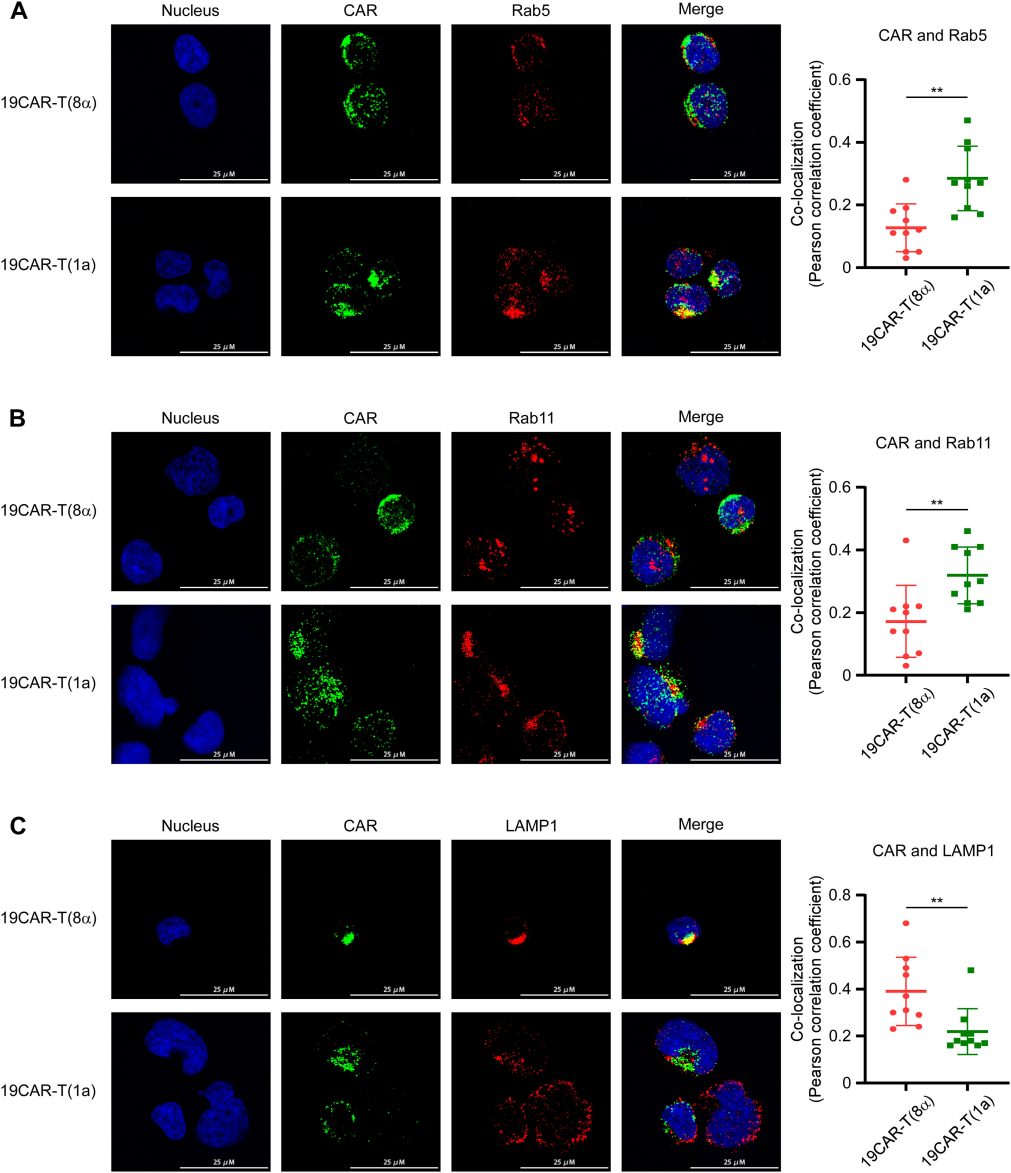
Internalized CD1a transmembrane CAR co-localizes more with early and recycling endosomes. **(A–C)** CAR-T cells were co-cultured with SEM cells at 1:1 for 4 h, and the cells were stained with mouse anti-FLAG antibody in combination with one of the following rabbit antibodies: anti-human Rab5 **(A)**, anti-human Rab11 **(B)**, or anti-human LAMP1 **(C)**. Then Goat anti-Mouse IgG (H+L) Secondary Antibody, DyLight™ 650 was used to stain mouse anti-FLAG antibody, and Goat anti-Rabbit IgG (H+L) Highly Cross-Adsorbed Secondary Antibody, Alexa Fluor™ 568 was used to stain rabbit anti- human Rab5, Rab11, or LAMP1. Positive CAR-T cells were identified by staining with the anti-FLAG antibody. The co-localization of CAR with Rab5, Rab11, or LAMP1 was indicated by the Pearson correlation coefficient, calculated by ImageJ. Ten cells with CAR and Rab5, Rab11, or LAMP1 positive, which were used to calculate the Pearson correlation coefficient were selected from three independent experiments. Two-tailed Student *t*-test, ** for P < 0.01. Error bars reflect ± SD.

Next, due to the 19CAR(1a) showing a rapid internalization rate, we explored whether the degradation of CAR would be influenced by TMD. We treated resting and activated CAR-T cells with cycloheximide to inhibit protein synthesis and observed that 19CAR(8α) underwent more degradation than 19CAR(1a) in both conditions (**Figures 2G, H**), suggesting the TMD influence the CAR degradation, likely due to the difference in the localization of CAR molecules post-internalization. CD1a molecule has been demonstrated to co-localize with early and recycling endosomes after internalization (25, 26). Here, we co-cultured CAR-T cells with SEM and explored the co-localization of early endosome marker Rab5 and recycling endosome marker Rab11 (27, 28) with CAR by immunofluorescence microscopy analysis. The results showed that more internalized 19CAR(1a) co-localized with Rab5 and Rab11 (**Figures 3A, B**), explaining the result that 19CAR-T(1a) had a rapid recycling rate. In contrast, more internalized 19CAR(8α) co-localized with LAMP1, a lysosome marker (26), indicating the fact that more CAR molecules were degraded in 19CAR-T(8α) compared to 19CAR-T(1a) (**Figures 3C**). In addition, these findings were further corroborated by similar experiments conducted in CAR-J cells, which yielded consistent results (**Supplementary Figure S4**). In summary, these data suggest that internalized CD1a transmembrane CAR co-localizes more with early and recycling endosomes, which explains the rapid recycling rate observed in 19CAR-T(1a) cells.

### 3.5 CD1a transmembrane CAR-T cells exhibit low activation levels and reduced exhaustion markers

**Figure 4:**
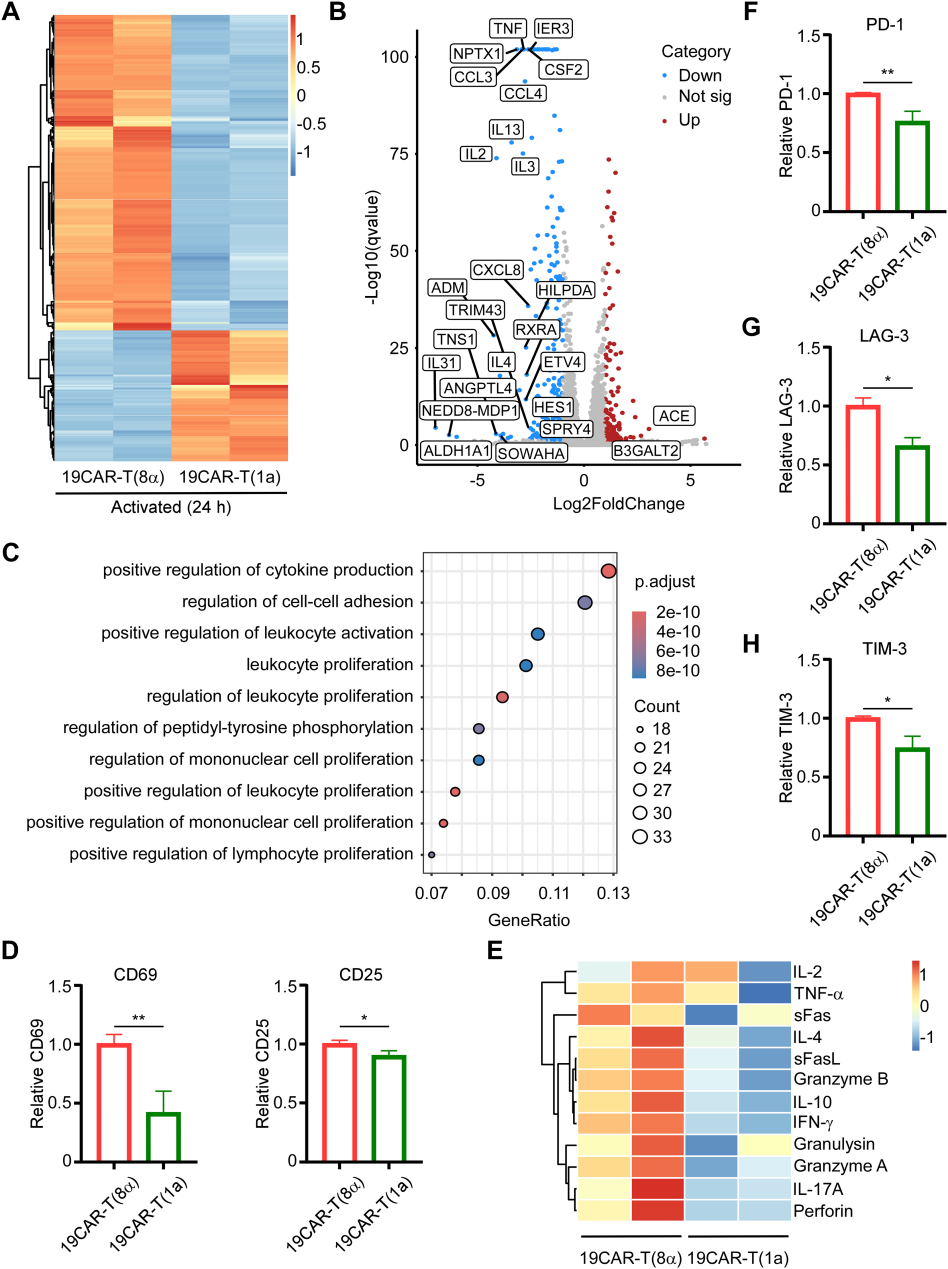
CD1a transmembrane CAR-T cells exhibit low activation levels and reduced exhaustion markers. **(A)** CAR-T cells were sorted after co-culture with SEM cells at 1:1 for 24 h, and then RNA-seq was performed. The heatmap of DEGs between 19CAR-T(1a) and 19CAR-T(8α) selecting based on |log2FoldChange| > 1 and Q-value < 0.05 were shown. **(B)** The volcano plot of DEGs between 19CAR-T(1a) and 19CAR-T(8α). The cutoff was set based on |log2FoldChange| > 1 and Q-value < 0.05. Some significant up or down regulation genes were marked. **(C)** Pathway analysis by Gene Ontology (GO) shows the top 10 pathways of the Biological Process of downregulated DEGs. **(D)** CAR-T cells were co-cultured with SEM at 1:1 for 24 h Then CD69 and CD25 were detected as an indication of CAR-T activation. Normalized to 19CAR-T(8α) expression of CD69 or CD25. **(E)** CAR-T cells were co-cultured with SEM cells at a 1:1 ratio for 24 h, and the supernatant was collected for detection of cytokines by LEGENDplex™ Human CD8/NK Panel. **(F, G)** CAR-T cells were co-cultured with SEM cells at 1:1 for 3 days, and exhaustion markers on CAR-T cells were detected by anti-human CD279 (PD-1) antibody **(F)**, anti-human CD223 (LAG-3) antibody **(G)**, and anti-human CD366 (TIM-3) antibody **(H)**. Normalized to 19CAR-T(8α) expression of exhaustion markers MFI. Two-tailed Student *t*-test, * for P < 0.05, ** for P < 0.01. Error bars reflect ± SD of three independent experiments. The RNA-seq analysis and CAR-T cytokines release detection were performed with two independent experiments.

Next, given the different surface CAR expression levels, we further investigated whether the CAR-T cell activation could be altered by CD1a transmembrane CAR. The CAR-T cells were sorted after co-culture with SEM cells at 1:1 for 24 h, followed by RNA sequencing (RNA-seq). The heatmap of differentially expressed genes (DEGs) showed that after co-culture, CAR-T cells were all activated and most of the DEGs were upregulated (**Supplementary Figure S5A**). Furthermore, the Venn diagram revealed that 19CAR-T(1a) had distinct transcriptomes from 19CAR-T(8α) after activation, among which 1920 DEGs were unique in 19CAR-T(1a) (**Supplementary Figure S5B**). In addition, a direct comparison of DEGs between 19CAR-T(1a) and 19CAR-T(8α) showed that most DEGs were downregulated in 19CAR-T(1a) (**Figure 4A**). Then, we used a volcano plot to show the signally upregulated or downregulated DEGs in 19CAR-T(1a) compared to 19CAR-T(8α) after activation. Notably, the cytokine genes IL2, IL3, IL4, and IL13 were significantly downregulated in 19CAR-T(1a) (**Figure 4B**). Pathway analysis of downregulated DEGs in 19CAR-T(1a) revealed different enriched pathways, such as positive regulation of cytokine production, positive regulation of leukocyte activation, cytokine-cytokine receptor interaction and so on (**Figure 4C**; **Supplementary Figure S5C**), suggesting the lower activation levels in 19CAR-T(1a). This was further supported by a reduction in CD69 and CD25, T cell activation markers (29), in 19CAR-T(1a) after 24 h of co-culture with SEM cells at a 1:1 ratio (**Figure 4D**). In addition, the 12 cytokines analysis also showed lower production of IL-4, IL-10, and IFNγ in 19CAR-T(1a) (**Figure 4E**), confirming its reduced activation levels. Previous study reveals that the CAR-T cell activation levels are associated with exhaustion (8). Thus, we co-cultured CAR-T cells and SEM cells at 1:1 for 72 h and then the exhaustion markers PD-1, LAG-3, and TIM-3 were detected (30). We found that these exhaustion markers on 19CAR-T(1a) were all lower than 19CAR-T(8α) (**Figures 4F–H**), suggesting the reduced exhaustion in 19CAR-T(1a).

Next, to ensure that these results were independent of the target cells, we constructed another target cell, CD19-K562, which possessed exogenously introduced CD19 proteins (**Supplementary Figure S6A**). First, we co-cultured CAR-T cells and CD19-K562 cells at different E/T ratios, and surface CAR on CAR-T cells was detected. The results were the same as the CAR-T cells co-cultured with SEM cells (**Supplementary Figure S6B**). Most importantly, 19CAR-T(1a) still showed lower CD69 level than 19CAR-T(8α) after co-culture with CD19-K562 cells (**Supplementary Figure S6C**), suggesting the lower activation levels in 19CAR-T(1a). Furthermore, the exhaustion markers PD-1, LAG-3, and TIM-3 on 19CAR-T(1a) were all lower than 19CAR-T(8α) after co-culture with CD19-K562 cells at different E/T ratios (1:2, 1:4 or 1:8) for 72 h, which was the same as the results on SEM cells (**Supplementary Figure S6D**). In summary, these data reflect that CD1a transmembrane CAR-T cells exhibit reduced activation and exhaustion levels, and these results are due to the CAR TMD rather than target cells. The high production of cytokines may result in CRS (3), and lower cytokine production in 19CAR-T(1a) could potentially alleviate CRS.

### 3.6 CD1a transmembrane CAR-T cells differentiate more into memory T cells

**Figure 5:**
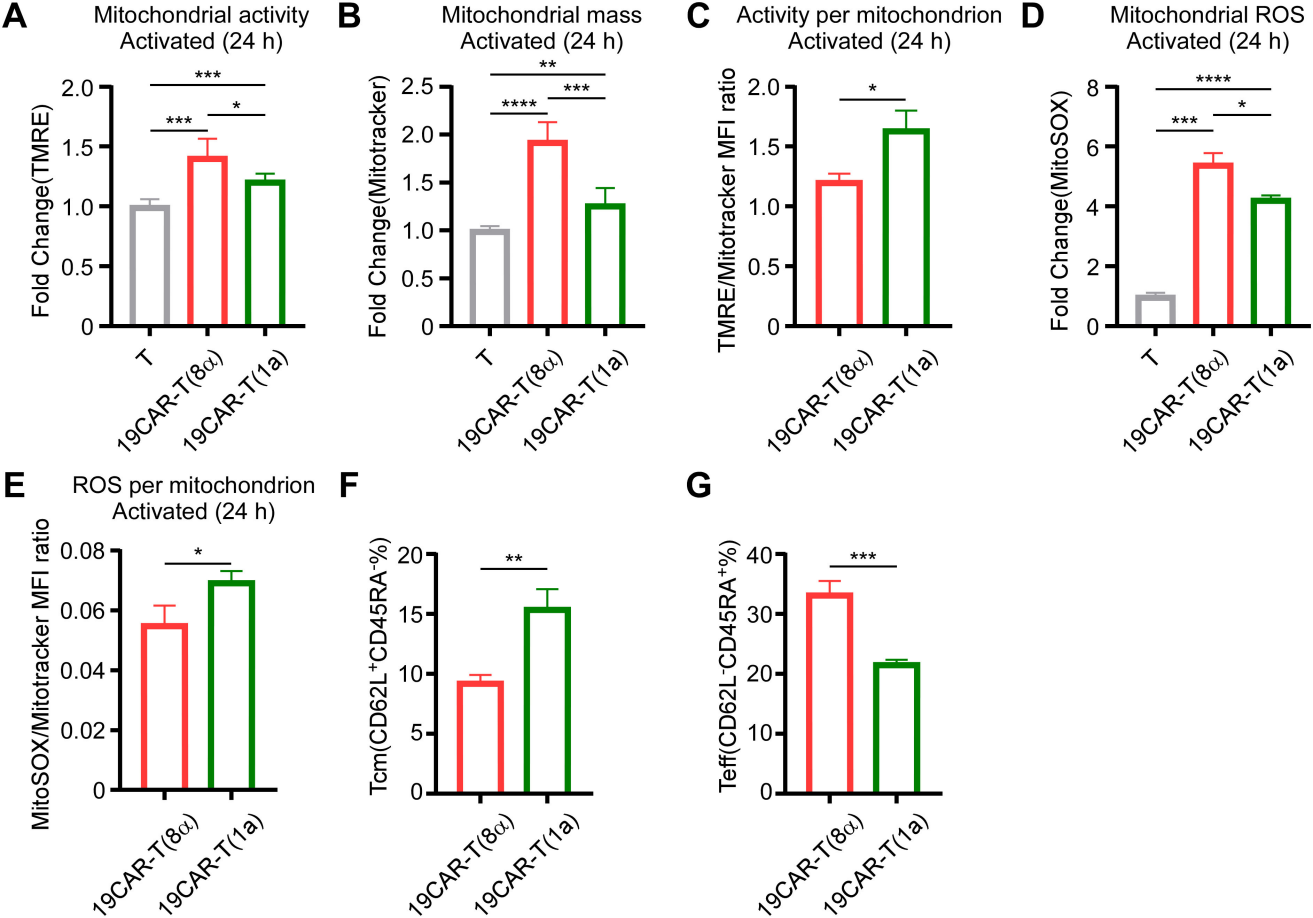
CD1a transmembrane CAR-T cells differentiate more into memory T cells. **(A)** CAR-T or T cells co-cultured with SEM at a 1:1 ratio for 24 h, and 200 nM TMRE was used to detect CAR-T cell mitochondrial activity. Normalized to T cells MFI of TMRE. **(B)** CAR-T or T cells were co-cultured with SEM at a 1:1 ratio for 24 h, and 200 nM Mitotracker was used to detect CAR-T cell mitochondrial mass. Normalized to T cells MFI of Mitotracker. **(C)** CAR-T cells TMRE/Mitotracker MFI ratio, respectively. **(D)** CAR-T or T cells were co-cultured with SEM at a 1:1 ratio for 24 h, and 5 μM MitoSOX was used to detect CAR-T cell mitochondrial ROS. Normalized to T cells MFI of MitoSOX. **(E)** CAR-T cells MitoSOX/Mitotracker MFI ratio, respectively. **(F, G)** CAR-T cells were co-cultured with SEM cells at 1:1 for 3 days, and CD62L or CD45RA on CAR-T cells were detected by anti-human CD62L or anti-human CD45RA antibodies. The percentage of Tcm (CD62L^+^CD45RA^-^) **(F)** and Teff (CD62L^-^CD45RA^+^) **(G)** were shown. Two-tailed Student *t*-test, * for P < 0.05, ** for P < 0.01, *** for P < 0.001, **** for P < 0.0001, the ns indicate no significant difference. Error bars reflect ± SD of three independent experiments.

Due to the different activation levels in 19CAR-T(8α) and 19CAR-T(1a) cells, we further explored whether the mitochondrial activity was influenced according to the research which suggested that mitochondrial function was associated with CAR-T cell function (31–33). Therefore, we measured the mitochondrial activity of these two CAR-T cells by TMRE and mitochondrial mass by using Mitotracker. We found that there was no significant difference in mitochondrial activity or mass between these two CAR-T cells at resting status (**Supplementary Figures S7A, B**). After short-time activation (4 h), both mitochondrial activity and mass increased, but no differences were observed between 19CAR-T(8α) and 19CAR-T(1a) (**Supplementary Figures S7C, D**). However, following long-time activation (24 h), both mitochondrial activity and mass were higher in 19CAR-T(8α) compared to 19CAR-T(1a) (**Figures 5A, B**), suggesting the higher activation level in 19CAR-T(8α), according to the study that T cell activation could increase mitochondrial mass (34). Here, we used the per-sample ratio of the TMRE/Mitotracker mean fluorescent index (MFI) as an indicator of activity per mitochondrion (35). The results reflected that 19CAR-T(1a) had higher mitochondrial activity per mitochondrion after both short- and long-time activation, although no difference was observed at resting status, suggesting the increased activity per mitochondrion was due to the CAR-T cell activation (**Figure 5C**; **Supplementary Figures S7E, F**). In addition, we also measured the mitochondrial ROS by flow cytometry using MitoSOX. Similar to TMRE, no significant differences in ROS levels between these two CAR-T cells were observed at resting status or after short-time activation (**Supplementary Figures S7G, H**). After long-time activation, these two CAR-T cells all had increased mitochondrial ROS levels than T cells, with 19CAR-T(8α) displaying higher ROS levels, also suggesting the high activation level in 19CAR-T(8α) (**Figure 5D**). Similarly, the ROS level per mitochondrion was indicated by using MitoSOX/Mitotrcker MFI ratio. Interestingly, 19CAR-T(1a) showed higher ROS per mitochondrion after long-time activation (**Figure 5E**; **Supplementary Figures S7I–J**). Previous studies have shown that increased mitochondrial activity correlated with a higher population of memory CAR-T cells (36). Thus, we detected memory CAR-T cells markers CD62L and CD45RA (37) after co-culture CAR-T cells with SEM at 1:1 for 72 h and found that 19CAR-T(1a) cells differentiated more into central memory CAR-T cells (Tcm, CD62L^+^CD45RA^-^) (**Figure 5F**). At the same time, 19CAR-T(1a) cells had fewer population of effector T cells (Teff, CD62L^-^CD45RA^+^) compared to 19CAR-T(8α) after 72 h of activation (**Figure 5G**). Collectively, our data indicate that CD1a transmembrane CAR-T cells have increased mitochondrial activity per mitochondrion upon activation and differentiate more into memory T cells, which may contribute to better clinical outcomes, as previously reported (35).

### 3.7 CD1a transmembrane CAR-T cells exhibit superior anti-tumor ability *in vivo*

**Figure 6:**
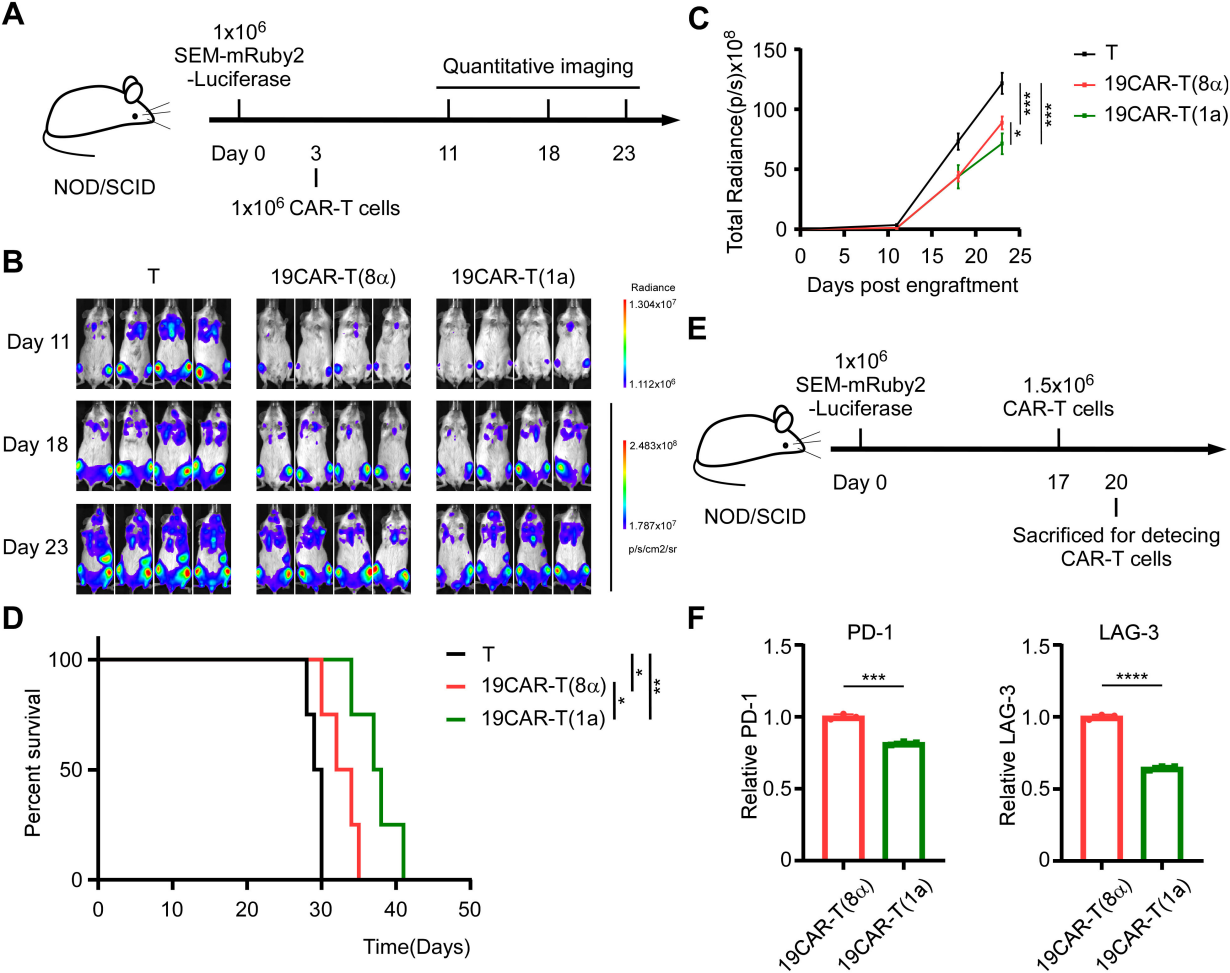
CD1a transmembrane CAR-T cells exhibit superior anti-tumor ability *in vivo.* **(A)** Schematic of mouse survival assay. **(B)** Representative bioluminescence images of tumor growth over time. **(C)** Total radiances post tumor cell engraftment of different treatment groups. Two-tailed Student *t*-test, * for P < 0.05, *** for P < 0.001, Error bars reflect ± SD of four mice. **(D)** The survival curve of mice post-transplantation was shown. Mouse survival was monitored regularly and statistically analyzed using a long-rank test, * for P < 0.05, ** for P < 0.01. **(E)** Schematic of CAR-T detection *in vivo*. **(F)** The PD-1 and LAG-3 on CAR-T cells from bone marrow were shown. Normalized to MFI of exhaustion markers expression on 19CAR-T(8α). Two-tailed Student *t*-test, *** for P < 0.001, **** for P < 0.0001, Error bars reflect ± SD of three mice.

Here, given the lower activation level of 19CAR-T(1a) cells compared to 19CAR-T(8α) cells *ex vivo*, it was important to assess the anti-tumor efficacy of 19CAR-T(1a) cells *in vivo*. By injecting SEM-mRuby2-Luciferase tumor cells into NOD/SCID mice intravenously, we constructed an SEM xenograft model. After tumor cells injection for 3 days, T cells, 19CAR-T(1a) cells, and 19CAR-T(8α) cells were injected, respectively. Tumor growth was monitored by the quantitative imaging system (**Figure 6A**). We found that the tumor progression could be delayed by 19CAR-T(1a) treatment (**Figures 6B, C**). Survival analysis revealed that both 19CAR-T(1a) and 19CAR-T(8α) cells prolonged mouse survival, with 19CAR-T(1a) treatment leading to significantly better outcomes (**Figure 6D**). In addition, mice were sacrificed on the third day after injection of CAR-T cells (**Figure 6E**). Interestingly, the exhaustion markers PD-1 and LAG-3 were all lower on 19CAR-T(1a) cells, consistent with *in vitro* results (**Figure 6F**). Taken together, these data suggest that 19CAR-T(1a) cells have a superior anti-tumor ability *in vivo*, and CD1a TMD could reduce CAR-T cell exhaustion.

## 4 Discussion

**Figure 7:**
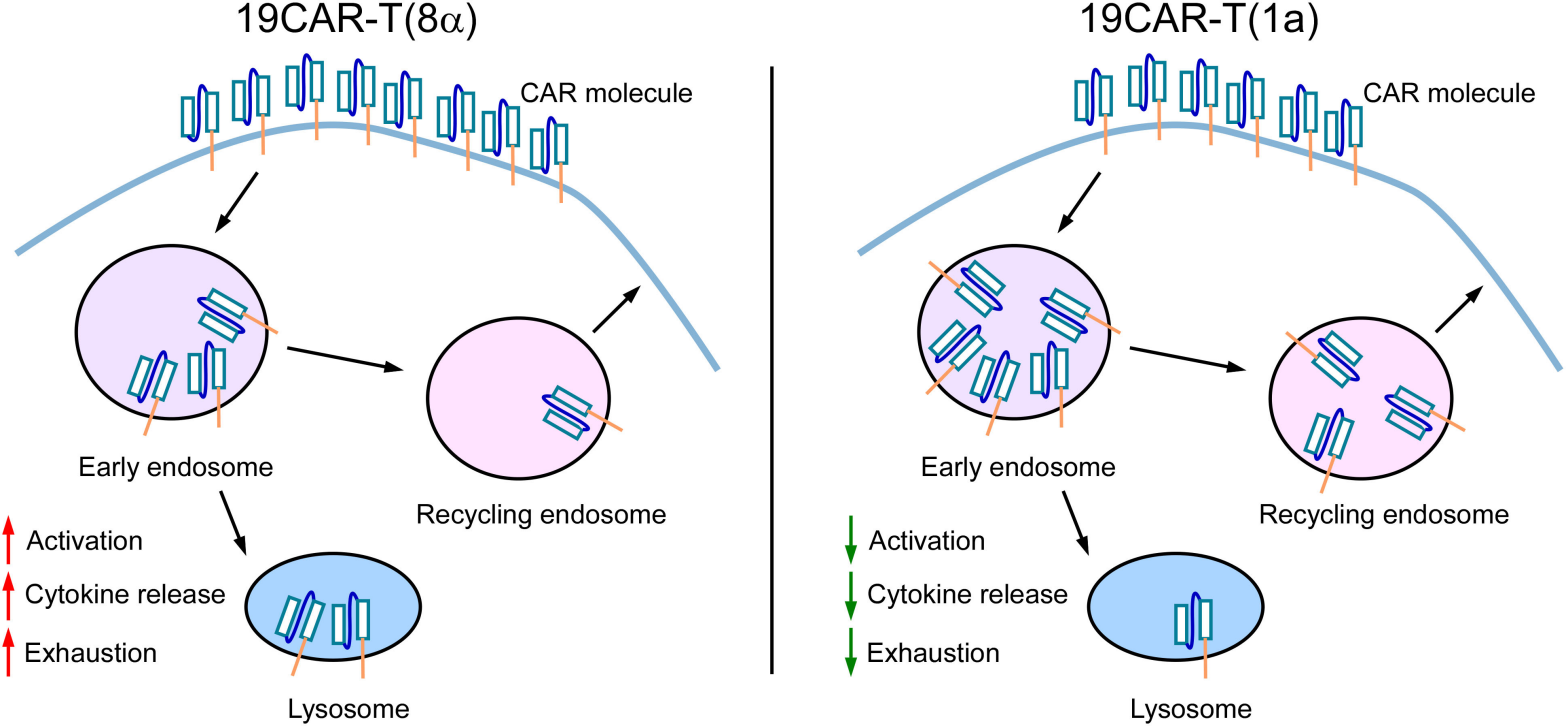
The Schematic for the mechanism of dynamic change of CD1a transmembrane CAR. 19CAR-T(1a) has lower surface CAR expression than 19CAR-T(8α). Internalized 19CAR(1a) co-localizes with early and recycling endosomes more than 19CAR(8α) and co-localizes with lysosomes less than 19CAR(8α). This phenotype results in lower activation levels, less cytokine release, and reduced exhaustion markers in 19CAR-T(1a) cells.

The CAR molecule is the most critical component of CAR-T cells, as it determines antigen recognition and affinity, thereby influencing CAR-T cell function (38). In this study, we engineered 19CAR-T(1a) cells with a CD1a TMD, which exhibited lower surface CAR expression compared to CD8α TMD CAR-T cells. Our findings revealed that internalized 19CAR(1a) co-localized more with early and recycling endosomes, while 19CAR(8α) showed greater co-localization with lysosomes. This phenotype resulted in lower activation levels, less cytokine release, and reduced exhaustion markers in 19CAR-T(1a) cells (**Figure 7**). Previous study has demonstrated that CAR-T cells with high surface CAR expression are associated with worse clinical outcomes in hematological malignancies (8), emphasizing the importance of density of CAR. Hence, our low surface expression CAR-T cells may offer better therapeutic outcomes. In addition, a similar study has been explored by fusing CTLA-4 tail to CAR structure, which endows CAR with different internalization and recycling rates (11). However, our strategy uniquely alters only the TMD, without introducing additional domains, distinguishing it from previous designs.

The commonly used TMDs in CAR constructs are derived from CD8 and CD28 molecules. Several studies demonstrate that these two different TMDs have different impacts on CAR-T cell function. CAR-T cells with CD8α hinge and TMD produce lower cytokine levels (13), and CD28 hinge and TMD could lower the threshold for CD19 CAR-T activation (39). The difference resulted from the reason that CD28 TMD could modulate CAR-T cell activities by engaging endogenous CD28 to influence CAR signaling (14), emphasizing the influence of TMD on CAR-T functions. Furthermore, research on CARs incorporating CD4, CD8, CD28, and CD3ζ TMDs demonstrated that TMDs regulated CAR surface expression levels and stability (40). Our study supported these findings by showing that the CD1a TMD lower surface CAR levels in 19CAR-T(1a) cells, further emphasizing the regulatory role of the TMD in CAR function. Due to the specificity of the CD1a molecule, the endocytosis is controlled by TMD (19), and we insert CD1a TMD into CAR. This design combines the endocytic properties of the CD1a TMD with CAR, as confirmed by internalization, recycling, and immunofluorescence assays. However, the same as the CD1a molecule, the precise mechanism by which the CD1a TMD controls CAR internalization remains unclear.

How to avoid severe CRS is another challenge in CAR-T therapy (3, 4). Here, our study revealed that 19CAR-T(1a) cells released fewer cytokines upon activation, indicating that TMD can influence CAR-T cell activation. Furthermore, according to the study that the CAR-T cell activation levels were associated with exhaustion (8), we detected the exhaustion markers on CAR-T cells and found that 19CAR-T(1a) cells exhibited lower exhaustion markers than 19CAR-T(8α) cells. These findings suggest that modifying the TMD could help reduce CRS and CAR-T cell exhaustion. Despite these promising results, several questions remain. First, according to the GFP signal and total CAR expression in CAR-T cells, 19CAR-T(1a) cells seem to express fewer CAR proteins than 19CAR-T(8α), even though both were infected with the same virus MOI. The reason behind this discrepancy in CAR expression levels is still unknown. Second, the CAR with CD1a TMD demonstrated a higher recycling rate compared to the CAR with CD8α, suggesting that CD1a recycling may also be regulated by its TMD, similar to its endocytosis has previously been shown to be controlled by the TMD (19). However, whether the recycling of CD1a is also controlled by TMD needs further exploration. Next, although 19CAR-T(1a) cells exhibit a superior anti-tumor ability and the CD1a TMD could reduce CAR-T cell exhaustion *in vivo*, 19CAR-T(1a) cells did not extend survival of mice by more than one week compared to 19CAR-T(8α) cells. As the tumor growth in mice was shown, the tumor growth accelerated with time, and these two CAR-T cells only slowed tumor progression. This is likely because SEM is an MLL-fusion malignancy, which progresses rapidly and easily to induce CAR-T cell exhaustion. Therefore, the CD1a TMD should be tested in other CAR types or different tumor models in future studies. In addition, further optimization of the TMD is necessary. CD1a, CD8, and CD28 are all endogenous proteins and an interesting study on *de novo* designed programmable membrane proteins incorporating TMDs from these molecules demonstrated reduced inflammatory cytokine release (41), highlighting the potential of TMD design in improving clinical outcomes.

## Data Availability

The data presented in the study are deposited in the Sequence Read Archive (SRA) repository, accession number PRJNA1222006.
