# Supplementary material for: Improved CAR internalization and recycling through transmembrane domain optimization reduces CAR-T cytokine release and exhaustion
Source: Front Immunol. 2025 Mar 27;16:1531344. doi: 10.3389/fimmu.2025.1531344 (PMC11983635; doi:10.3389/fimmu.2025.1531344)
Supplement: Supplementary file 1 [file DataSheet1.docx]

**Supplementary figures**


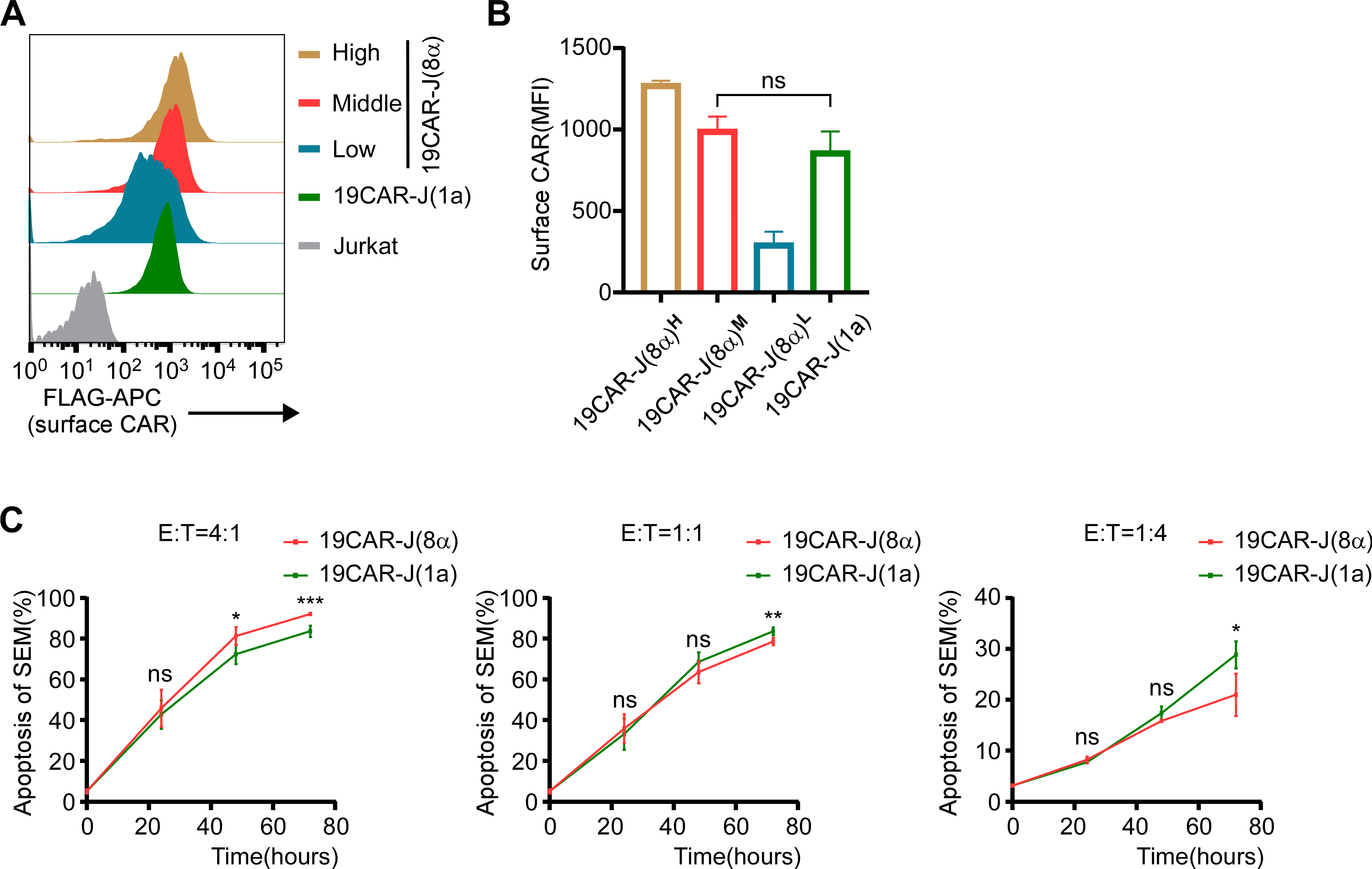


**Figure. S1 19CAR-J(1a) and 19CAR-J(8α) cells with similar surface CAR expression levels have similar killing effects**

**(A)** The surface CAR expression on CAR-J cells indicated by anti-FLAG antibody. **(B)** The MFI of surface CAR expression on CAR-J cells. **(C)** CAR-J cells were co-cultured with SEM cells at 4:1, 1:1, or 1:4 for 24 h, 48 h, or 72 h. The apoptosis of SEM cells was detected by using the Annexin V kit. Two-tailed Student *t*-test, * for P < 0.05, ** for P < 0.01, *** for P < 0.001, the ns indicate no significant difference. Error bars reflect ± SD of three independent experiments.


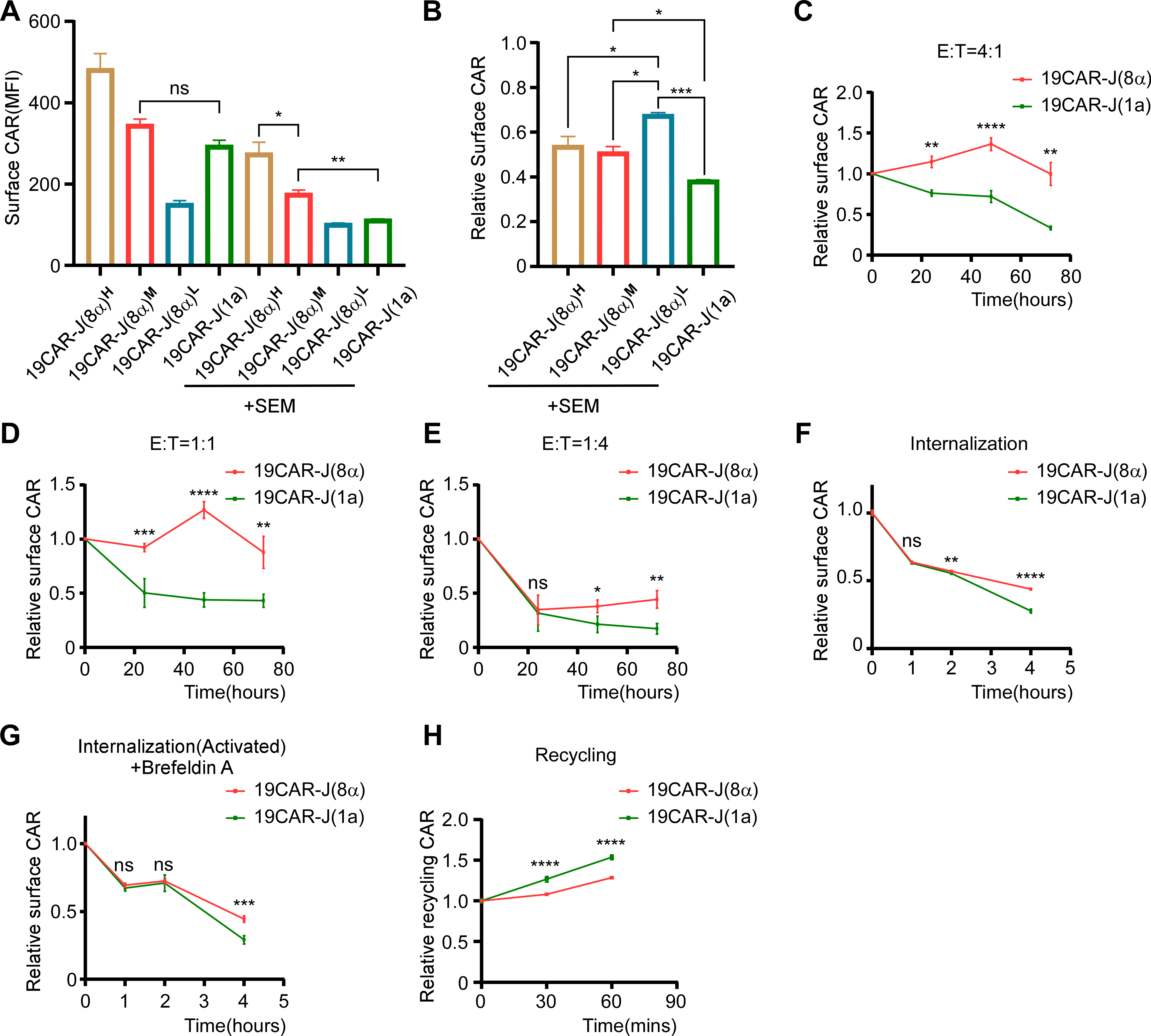


**Figure. S2 CD1a transmembrane CAR shows rapid internalization and recycling rate on CAR-J cells**

**(A**) CAR-J cells with different surface CAR expression levels were co-cultured with or without SEM cells at 1:2 for 24 h. The MFI of surface CAR expression on CAR-J cells. **(B)** CAR-J cells with different surface CAR expression levels were co-cultured with SEM cells at 1:2 for 24 h. Normalized to each CAR-J cell non-cocultured surface CAR, respectively. **(C-E)** 19CAR-J(8α)^M^ and 19CAR-J(1a) cells were co-cultured with SEM cells at 4:1**(C)**, 1:1**(D)**, or 1:4**(E)** for 24, 48, or 72 h. The surface CAR on CAR-J cells was detected by anti-FLAG antibody. Normalized to each CAR-J cell non-cocultured surface CAR, respectively. **(F)** Antibody-based assay for CAR internalization. CAR-J cells were stained with anti-FLAG Antibody at 4°C for 30 min. Then these cells were incubated at 37°C for 0, 1, 2, or 4 h in the medium. Next, these cells were stained with goat anti-rat IgG secondary antibody at 4°C for 30 min. The decrease in the percentage of cells staining positive for the secondary antibody was quantified by flow cytometry as an indication of the internalization rate. Normalized to the percentage of each CAR-J cell staining positive for the secondary antibody non-incubated at 37°C, respectively. **(G)** Brefeldin A-based assay for CAR internalization. CAR-J cells were co-cultured with SEM at a 1:1 ratio for 0, 1, 2, or 4 h with the presence of 10 μM Brefeldin A, the surface CAR was detected by anti-FLAG antibody. Normalized to each CAR-J cell Brefeldin A non-treated surface CAR, respectively. **(H)** Antibody-based assay for CAR recycling. CAR-J cells were stained with anti-FLAG Antibody at 37°C for 30 min. Then the cells were stained with goat anti-rat IgG secondary antibody at 4°C for 30 min (baseline) or at 37°C for 30 min or 60 min. The increase in the percentage of cells staining positive for the secondary antibody was quantified by flow cytometry as an indication of recycling. Two-tailed Student *t*-test, * for P < 0.05, ** for P < 0.01, *** for P < 0.001, **** for P < 0.0001, the ns indicate no significant difference. Error bars reflect ± SD of three independent experiments.


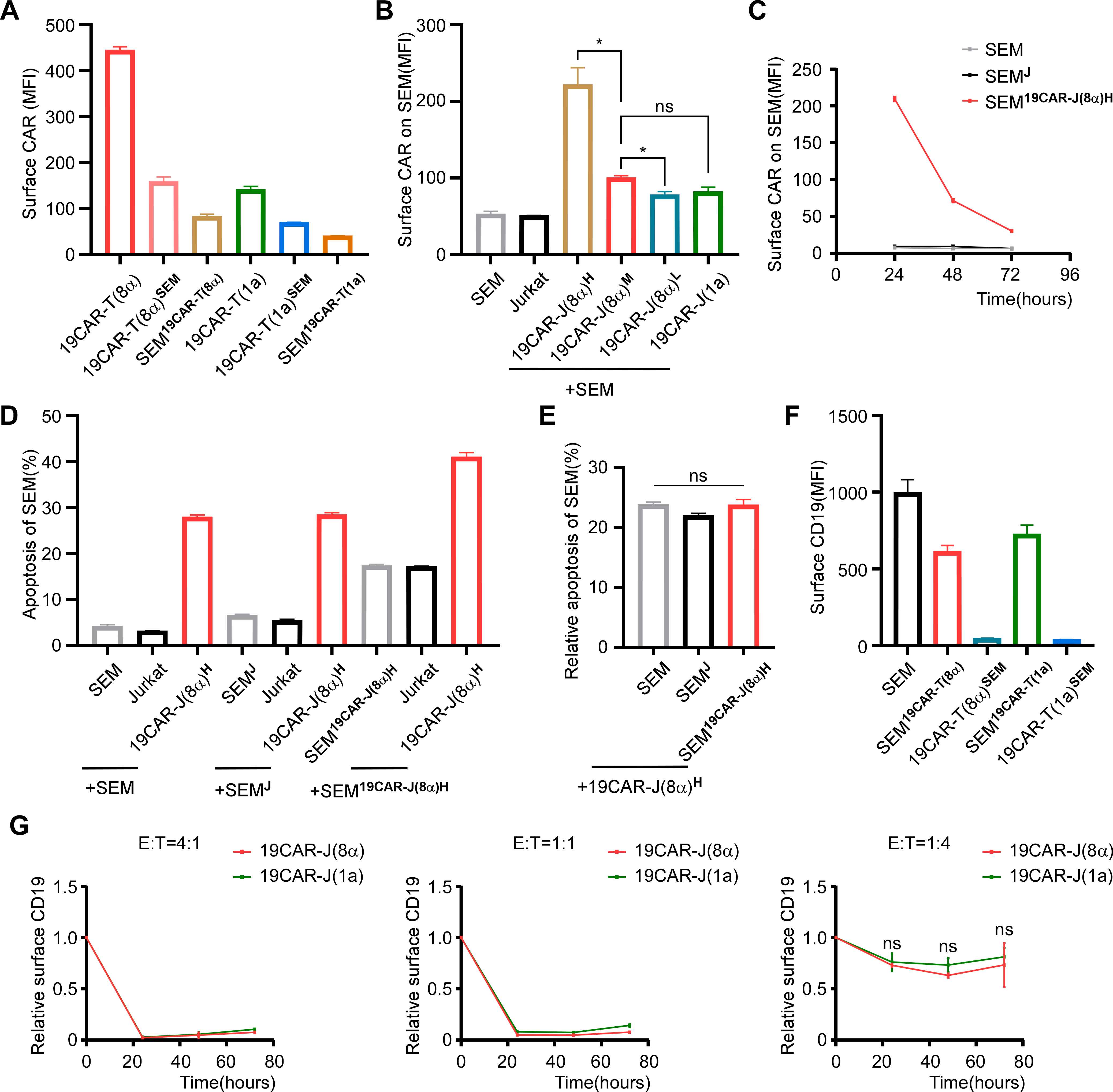


**Figure. S3 Transferred a small amount of CAR on SEM cells won't affect its apoptosis induced by CAR-J cells**

**(A**) CAR-T cells were co-cultured with or without SEM cells at 1:1 for 1 h. The MFI of surface CAR on CAR-T or SEM cells. 19CAR-T(8α)^SEM^ and 19CAR-T(1a)^SEM^: The CAR-T cells co-cultured with SEM; SEM^19CAR-T(8α)^ and SEM^19CAR-T(1a)^: The SEM cells co-cultured with CAR-T cells. **(B)** CAR-J cells with different surface CAR expression levels were co-cultured with SEM cells at 1:1 for 24 h. The transferred surface CAR on SEM cells was detected by anti-FLAG antibody. **(C)** The SEM cells were sorted after co-culture with 19CAR-J(8α)^H^ cells at 1:1 for 24 h, and the transferred surface CAR on SEM cells was detected by anti-FLAG antibody. SEM^J^: Sorted SEM cells co-cultured with Jurkat; SEM^19CAR-J(8α)H^: Sorted SEM cells co-cultured with 19CAR-J(8α)^H^. **(D)** SEM cells were sorted after co-culturing with Jurkat or 19CAR-J(8α)^H^ cells at 1:1 for 24 h. Then these sorted SEM cells co-cultured with 19CAR-J(8α)^H^ cells at 1:1 for another 24 h. The apoptosis of SEM cells was detected by using the Annexin V kit. **(E)** Relative apoptosis of SEM cells was indicated by co-cultured SEM apoptosis minus non-cocultured SEM apoptosis. **(F)** CAR-T cells were co-cultured with SEM cells at 1:1 for 1 h. The MFI of CD19 on CAR-T or SEM cells. **(G)** 19CAR-J(8α)^M^ and 19CAR-J(1a) cells were co-cultured with SEM cells at 4:1, 1:1, or 1:4 for 24, 48, or 72 h. The surface CD19 on SEM cells was detected by anti-CD19 antibody. Normalized to the non-cocultured SEM CD19. Two-tailed Student *t*-test, * for P < 0.05, the ns indicate no significant difference. Error bars reflect ± SD of three independent experiments.


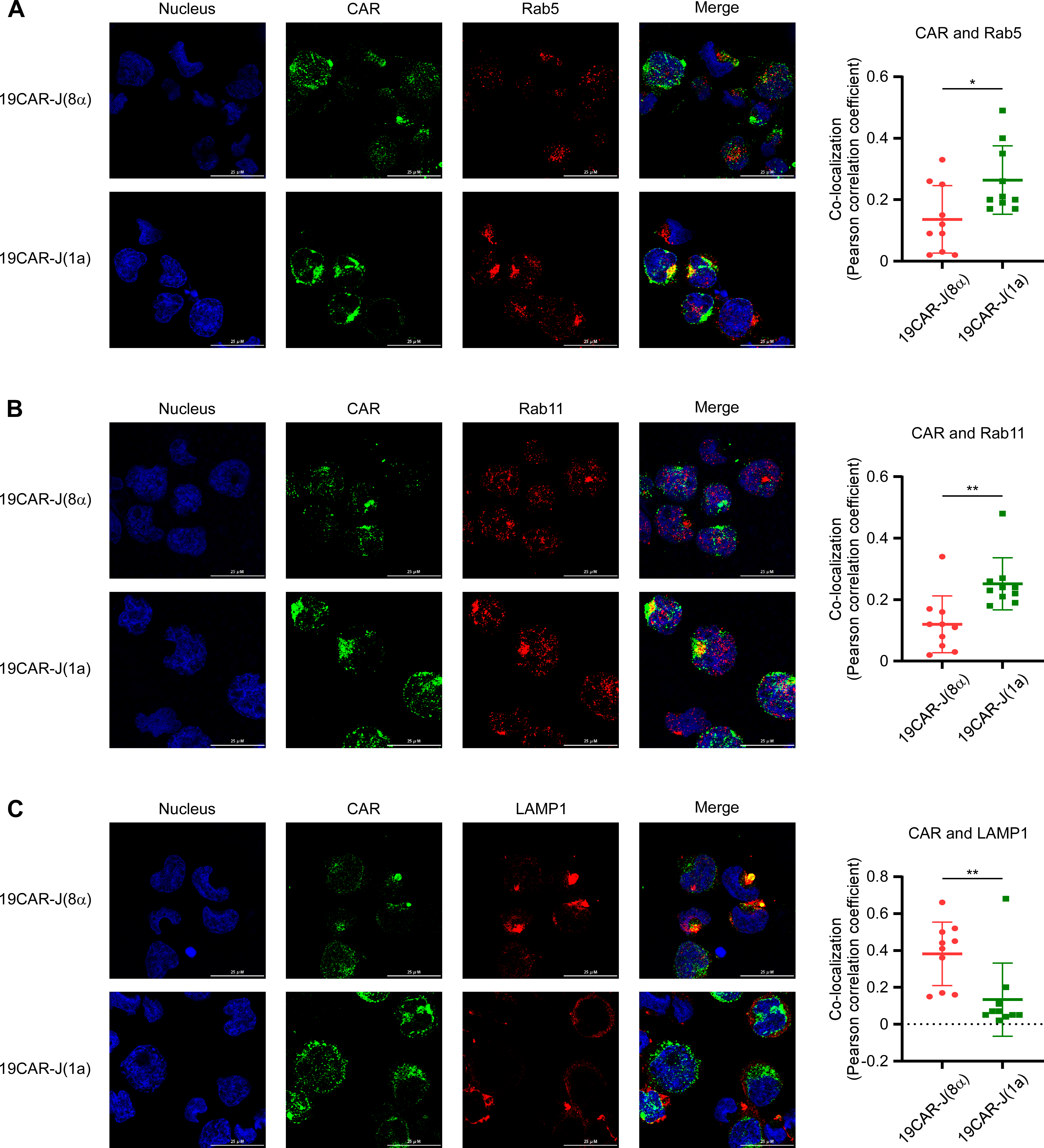


**Figure. S4 Internalized CD1a transmembrane CAR co-localizes with early and recycling endosomes in CAR-J cells**

**(A-C)** CAR-J cells were co-cultured with SEM cells at 1:1 for 4 h, and the cells were stained with mouse anti-FLAG antibody in combination with one of the following rabbit antibodies: anti-human Rab5 (**A**), anti-human Rab11 (**B**), or anti-human LAMP1 (**C**). Then Goat anti-Mouse IgG (H+L) Secondary Antibody, DyLight^TM^ 650 was used to stain mouse anti-FLAG antibody, and Goat anti-Rabbit IgG (H+L) Highly Cross-Adsorbed Secondary Antibody, Alexa Fluor^TM^ 568 was used to stain rabbit anti- human Rab5, Rab11, or LAMP1. Positive CAR-J cells were identified by staining with the anti-FLAG antibody. The co-localization of CAR with Rab5, Rab11, or LAMP1 was indicated by the Pearson correlation coefficient, calculated by ImageJ. Ten cells with CAR and Rab5, Rab11, or LAMP1 positive, which were used to calculate Pearson correlation coefficient were chose from three independent experiments. Two-tailed Student *t*-test, * for P < 0.05, ** for P < 0.01. Error bars reflect ± SD.


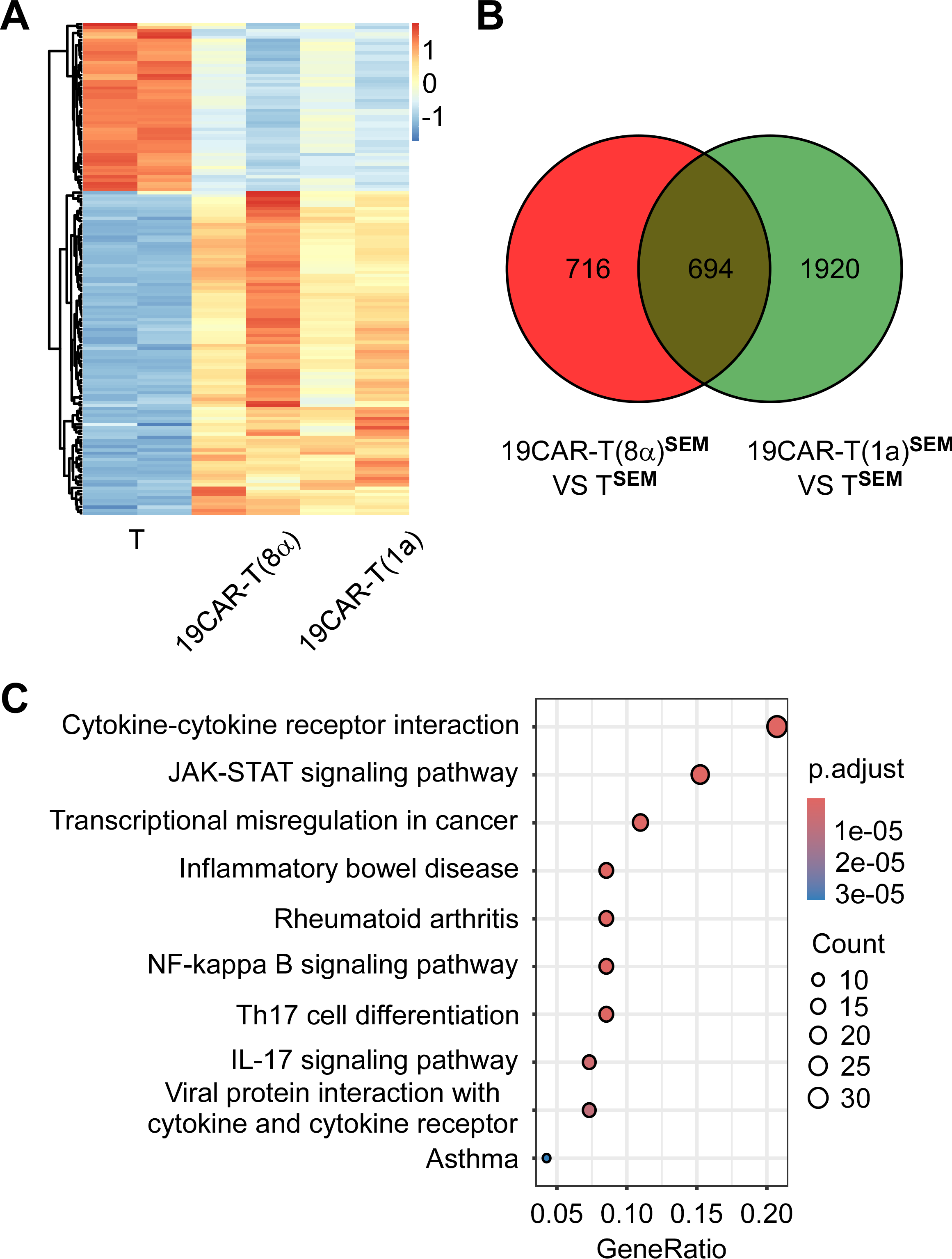


**Figure. S5 19CAR-T(1a) has a different transcriptome from 19CAR-T(8α)**

**(A)** CAR-T cells were sorted after co-culture with SEM cells at 1:1 for 24 h, and then RNA-seq was performed. The heatmap of differentially expressed genes (DEGs) between T, 19CAR-T(1a), and 19CAR-T(8α) selecting based on |log2FoldChange| > 1 and Q-value < 0.05 were shown. **(B)** A Venn diagram of DEGs between 19CAR-T(1a) vs T and 19CAR-T(8α) vs T was shown. **(C)** Pathway analysis by the Kyoto Encyclopedia of Genes and Genomes (KEGG) shows the top 10 pathways of downregulated DEGs between 19CAR-T(1a) vs 19CAR-T(8α).


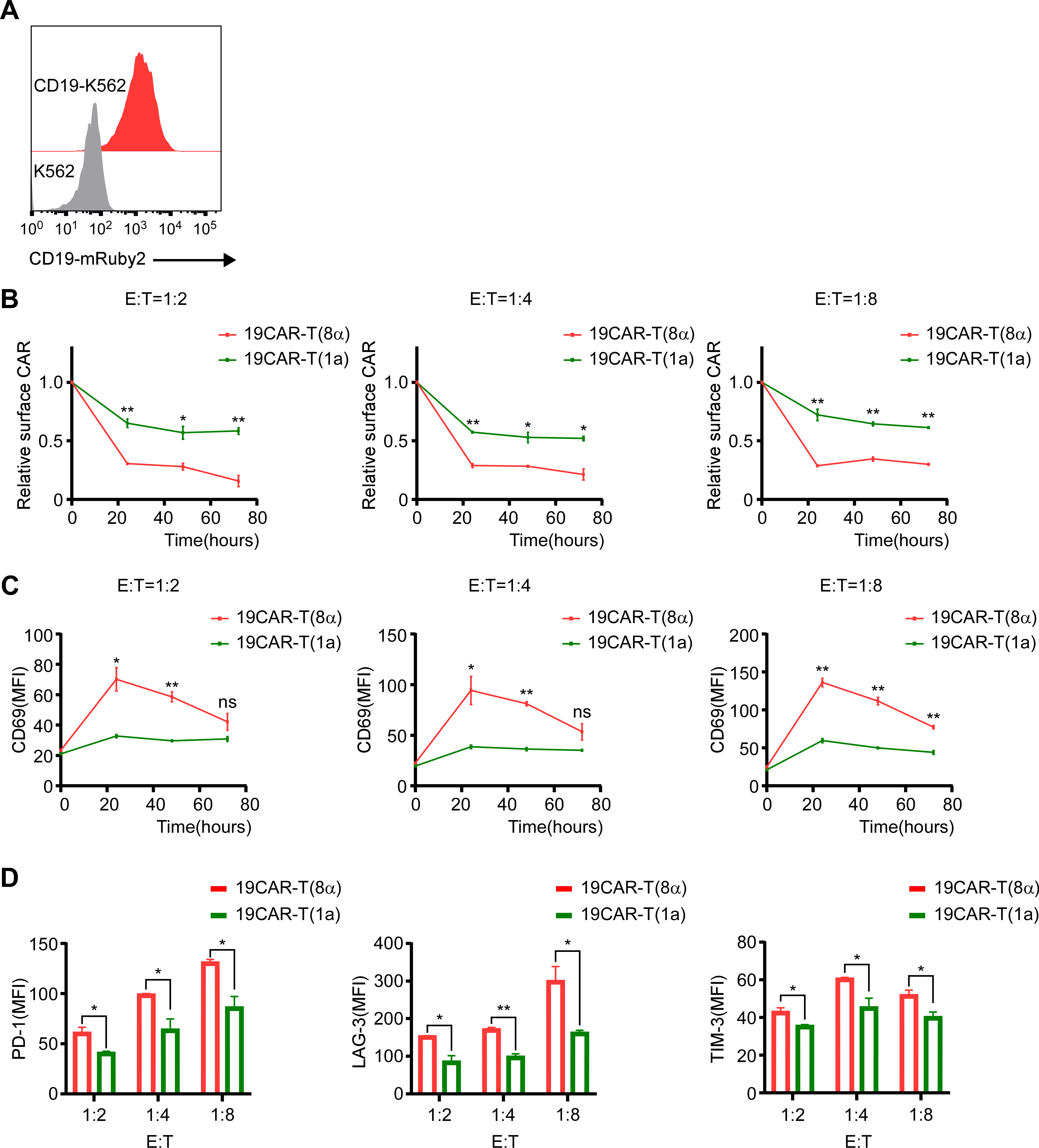


**Figure. S6 19CAR-T(1a) cells show low activation levels and reduced exhaustion markers co-culture with CD19-K562 cells**

**(A)** The positive rate of CD19-K562 was indicated by the mRuby2 signal. **(B)** CAR-T cells were co-cultured with CD19-K562 cells at 1:2, 1:4, or 1:8 for 24, 48 or 72 h. The surface CAR on CAR-T cells was detected by anti-FLAG antibody. Normalized to each CAR-T cell non-cocultured surface CAR, respectively. **(C)** CAR-T cells were co-cultured with CD19-K562 cells at 1:2, 1:4, or 1:8 for 24, 48 or 72 h. The CD69 on CAR-T cells was detected by anti-CD69 antibody. **(D)** CAR-T cells were co-cultured with CD19-K562 cells at 1:2, 1:4, or 1:8 for 3 days, and exhaustion markers on CAR-T cells were detected by anti-human CD279 (PD-1) antibody, anti-human CD223 (LAG-3) antibody, and anti-human CD366 (TIM-3) antibody. Two-tailed Student *t*-test, * for P < 0.05, ** for P < 0.01, the ns indicate no significant difference. Error bars reflect ± SD of three independent experiments.


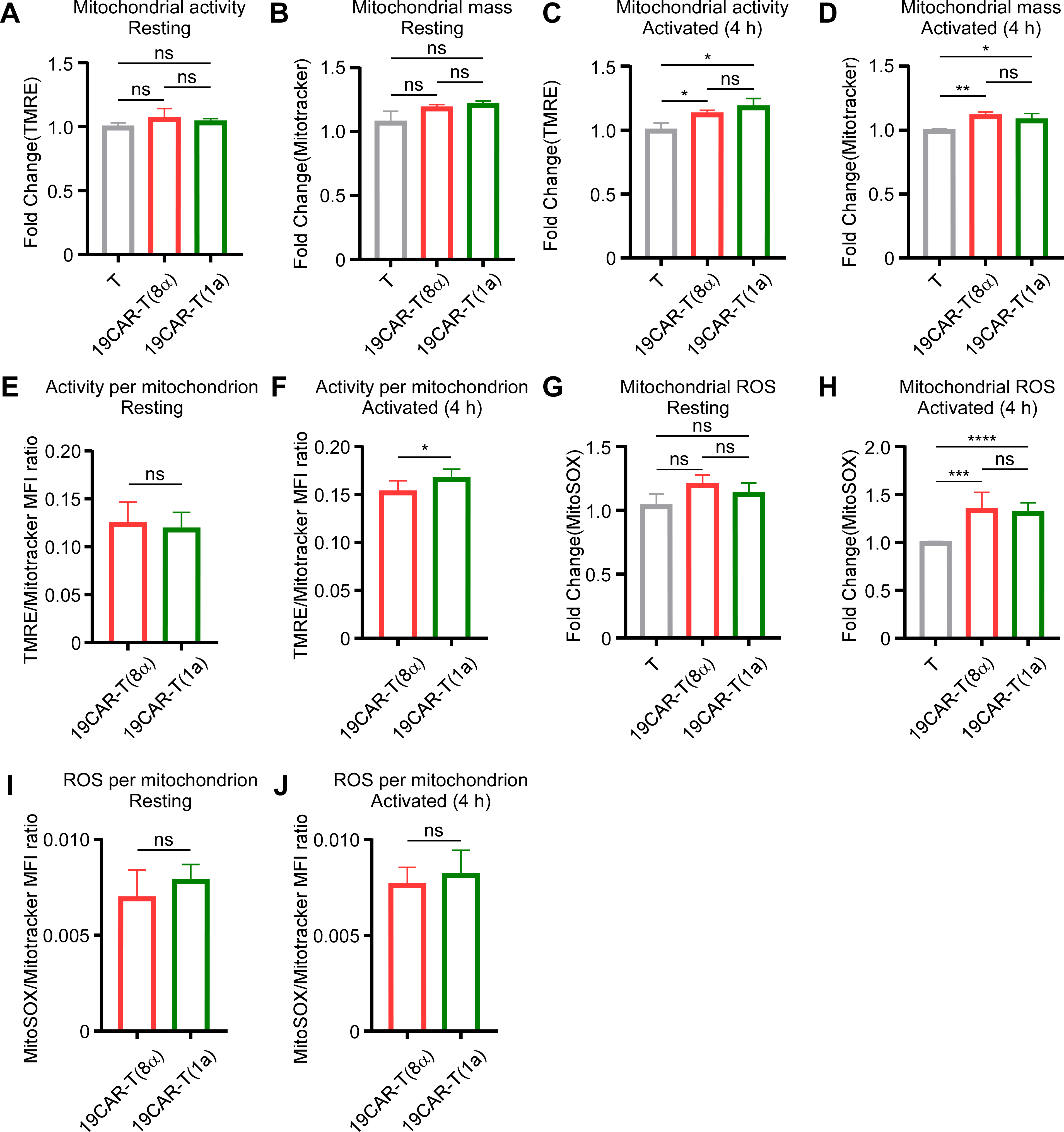


**Figure. S7 Mitochondrial activity/mass/ROS in resting or short-time activation CAR-T cells**

**(A)** 200 nM TMRE was used to detect CAR-T or T cell mitochondrial activity at resting status. Normalized to T cells MFI of TMRE. **(B)** 200 nM Mitotracker was used to detect CAR-T or T cell mitochondrial mass at resting status. Normalized to T cells MFI of Mitotracker. **(C)** CAR-T or T cells were co-cultured with SEM at a 1:1 ratio for 4 h, and 200 nM TMRE was used to detect CAR-T cell mitochondrial activity. Normalized to T cells MFI of TMRE. **(D)** CAR-T or T cells were co-cultured with SEM at a 1:1 ratio for 24 h, and 200 nM Mitotracker was used to detect CAR-T cell mitochondrial mass. Normalized to T cells MFI of Mitotracker. **(E)** CAR-T cells TMRE/Mitotracker MFI ratio at resting status, respectively. **(F)** CAR-T cells TMRE/Mitotracker MFI ratio at short-time activation status (4 h), respectively. **(G)** 5 μM MitoSOX was used to detect CAR-T or T cells mitochondrial ROS at resting status. Normalized to T cells MFI of MitoSOX. **(H)** CAR-T or T cells were co-cultured with SEM at a 1:1 ratio for 4 h, and 5 μM MitoSOX was used to detect CAR-T or T cells mitochondrial ROS. Normalized to T cells MFI of MitoSOX. **(I)** CAR-T cells MitoSOX/Mitotracker MFI ratio at resting status, respectively. **(J)** CAR-T cells MitoSOX /Mitotracker MFI ratio at short-time activation status (4 h), respectively. Two-tailed Student *t*-test, * for P < 0.05, ** for P < 0.01, *** for P < 0.001, **** for P < 0.0001, the ns indicate no significant difference. Error bars reflect ± SD of three independent experiments.
